# Supplementary material for: Bleeding complications of thromboprophylaxis with dabigatran, nadroparin or rivaroxaban for 6 weeks after total knee arthroplasty surgery: a randomised pilot study
Source: BMJ Open. 2021 Jan 18;11(1):e040336. doi: 10.1136/bmjopen-2020-040336 (PMC7813324; doi:10.1136/bmjopen-2020-040336)
Supplement: Supplementary data [file bmjopen-2020-040336supp001.pdf]

Naam: Geb.datum: Datum:

Knee injury and Osteoarthritis Outcome Score (KOOS)

Groot IB de, Favejee M, Reijman M, Verhaar JAN, Terwee CB.

Instructies: Deze enquête stelt vragen in verband met uw visie betreffende uw knie. Deze informatie helpt ons te achterhalen hoe u zich voelt en in hoeverre het mogelijk is voor u om uw dagelijkse activiteiten uit te voeren. Beantwoord de onderstaande vragen door **één** antwoord aan te vinken dat voor u van toepassing is. Als u niet geheel zeker bent van uw antwoord, graag toch het best mogelijke antwoord geven.

Belangrijk: Probeer uitsluitend antwoord met betrekking tot het kniegewricht waarvoor u in deze studie behandeld wordt.

Symptomen

Deze vragen dienen te worden beantwoord met betrekking tot de knie symptomen gedurende de afgelopen week.

	Nooit	Bijna nooit	Soms	Vaak	Altijd
**S1. Is uw knie gezwollen?**	O	O	O	O	O
**S2. Voelt u knarsen in uw knie of hoort u uw knie klikken of een ander geluid als u uw knie beweegt?**	O	O	O	O	O
**S3. Blijft uw knie steken of schiet uw knie op slot bij bewegen?**	O	O	O	O	O

	Altijd	Vaak	Soms	Bijna nooit	Nooit
**S4. Kan u uw knie volledig strekken?**	O	O	O	O	O
**S5. Kan u uw knie volledig buigen?**	O	O	O	O	O

Stijfheid

De volgende vragen betreffen de hoeveelheid gewrichtstijfheid die u ervaren heeft in uw knie gedurende de **afgelopen week.** Stijfheid is een gevoel van restrictie of traagheid in de gemakkelijkheid waarmee u uw kniegewricht kan bewegen.

	Niet	Mild	Gemiddeld	Ernstig	Extreem
**S6. Hoe ernstig is de stijfheid van uw kniegewricht als u wakker wordt 's morgens?**	O	O	O	O	O
**S7. Hoe ernstig is de stijfheid van uw kniegewricht nadat u gezeten, gelegen of gerust heeft later op de dag?**	O	O	O	O	O

**Pijn**	Nooit	Maandelijks	Wekelijks	Dagelijks	Altijd
**P1. Hoe vaak ervaart u kniepijn?**	O	O	O	O	O

Hoeveel kniepijn heeft u ervaren de **afgelopen week** tijdens de volgende activiteiten?

	Niet	Mild	Gemiddeld	Ernstig	Extreem

Naam: Geb.datum: Datum:

**P2. Draaien / roteren van uw knie**	O	O	O	O	O
**P3. Volledig strekken van de knie**	O	O	O	O	O
**P4. Volledig buigen van de knie**	O	O	O	O	O
**P5. Lopen op een vlak oppervlak**	O	O	O	O	O
**P6. Op- en neer lopen van een trap**	O	O	O	O	O
**P7. ‘s Nachts in bed**	O	O	O	O	O
**P8. Zitten of liggen**	O	O	O	O	O
**P9. Rechtop staan**	O	O	O	O	O

Functioneren, dagelijkse bezigheden

De volgende vragen betreffen uw fysieke functioneren. Hierbij wordt bedoeld de mogelijkheid om u te bewegen en voor uzelf te zorgen. Voor de volgende activiteiten graag aangeven wat de moeilijkheidsgraad is die u ervaren heeft gedurende de afgelopen week als gevolg van uw knie.

	**Niet**	**Mild**	**Gemiddeld**	**Ernstig**	**Extreem**
**A1. Aflopen van een trap**	O	O	O	O	O
**A2. Oplopen van een trap**	O	O	O	O	O
**A3. Opstaan nadat u gezeten heeft**	O	O	O	O	O
**A4. Staan**	O	O	O	O	O
**A5. Buigen om iets op te pakken**	O	O	O	O	O
**A6. Lopen op een vlak oppervlak**	O	O	O	O	O
**A7. In- en uit een auto stappen**	O	O	O	O	O
**A8. Boodschappen doen / winkelen**	O	O	O	O	O
**A9. Sokken / panty aandoen**	O	O	O	O	O
**A10. Opstaan uit bed**	O	O	O	O	O
**A11. Sokken / panty uitdoen**	O	O	O	O	O
**A12. In bed liggen (draaien, behouden van de knie positie)**	O	O	O	O	O
**A13. In- en uit bad stappen**	O	O	O	O	O
**A14. Zitten**	O	O	O	O	O
**A15. Naar het toilet gaan**	O	O	O	O	O

van der Veen L, *et al. BMJ Open* 2021; 11:e040336. doi: 10.1136/bmjopen-2020-040336

Naam: Geb.datum: Datum:

**A16. Zware huishoudelijke taken (verplaatsen van dozen, boenen van vloer, etc.)**	O	O	O	O	O
**A17. Lichte huishoudelijke taken (koken, stoffen, etc.)**	O	O	O	O	O

Functioneren, sporten, en ontspanningsactiviteiten

De volgende vragen betreffen uw fysieke functies wanneer u actief bent op een hoger niveau. De vragen dienen te worden beantwoord naar aanleiding van de moeilijkheidsgraad die u ervaart door uw knie gedurende de **afgelopen week**.

	**Niet**	**Mild**	**Gemiddeld**	**Ernstig**	**Extreem**
**SP1. Hurken**	O	O	O	O	O
**SP2. Rennen**	O	O	O	O	O
**SP3. Springen**	O	O	O	O	O
**SP4. Draaien / roteren van uw geblesseerde knie**	O	O	O	O	O
**SP5. Knielen / bukken**	O	O	O	O	O

Kwaliteit van leven

	**Nooit**	**Maandelijks**	**Wekelijks**	**Dagelijks**	**Altijd**
**Q1. Hoe vaak bent u zich bewust van uw knie probleem?**	O	O	O	O	O

	**Niet**	**Nauwelijks**	**Gemiddeld**	**Ernstig**	**Extreem**
**Q2. Heeft u uw levenswijze aangepast om potentiële schadelijke activiteiten aan u knie te vermijden?**	O	O	O	O	O
**Q3. In welke mate bent u gehinderd door het gemis van vertrouwen in uw knie?**	O	O	O	O	O

	**Niet**	**Mild**	**Gemiddeld**	**Ernstig**	**Extreem**
**Q4. In het algemeen, hoeveel hinder ervaart u door uw knie?**	O	O	O	O	O

Scoring: elk item wordt gescoord tussen 0 en 4 en de ruwe score van elke sectie is de som of de item scores. De score wordt daarna omgezet in een 0 -100 schaal. Een hogere score indiceert minder problemen

**Schaal**	**Ruwe score**	**Herberekende score**	**MDC90**
Pijn	/36	100 - Actueel ruwe score x 100 : Mogelijke ruwe score range	12 punten
Symptomen	/28		8 punten
ADL	/68		10 punten
Sport/Rec	/20		19 punten
QvL	/16		13 punten
