# Supplementary material for: Bleeding complications of thromboprophylaxis with dabigatran, nadroparin or rivaroxaban for 6 weeks after total knee arthroplasty surgery: a randomised pilot study
Source: BMJ Open. 2021 Jan 18;11(1):e040336. doi: 10.1136/bmjopen-2020-040336 (PMC7813324; doi:10.1136/bmjopen-2020-040336)
Supplement: Author's manuscript [file bmjopen-2020-040336.draft_revisions.pdf]

BMJ Open is committed to open peer review. As part of this commitment we make the peer review history of every article we publish publicly available.

When an article is published we post the peer reviewers' comments and the authors' responses online. We also post the versions of the paper that were used during peer review. These are the versions that the peer review comments apply to.

The versions of the paper that follow are the versions that were submitted during the peer review process. They are not the versions of record or the final published versions. They should not be cited or distributed as the published version of this manuscript.

BMJ Open is an open access journal and the full, final, typeset and author-corrected version of record of the manuscript is available on our site with no access controls, subscription charges or pay-per-view fees (http://bmjopen.bmj.com).

If you have any questions on BMJ Open's open peer review process please email info.bmjopen@bmj.com

Bleeding complications of thromboprophylaxis with dabigatran, nadroparin or rivaroxaban for six weeks after total knee arthroplasty surgery – a randomised pilot study

Journal:	*BMJ Open*
Manuscript ID	bmjopen-2020-040336
Article Type:	Original research
Date Submitted by the Author:	11-May-2020
Complete List of Authors:	van der Veen, Lucia; Martini Hospital, Clinical Pharmacy; Ommelander Hospital Groningen, Department of Clinical Pharmacy<br>Segers, Marijn; Martini Hospital, Department of Clinical Pharmacy and Toxicology<br>van Raay, Jos; Martini Hospital Groningen, Department of Orthopaedic Surgery<br>Gerritsma-Bleeker, Carina; Martini Hospital Groningen, Department of Orthopaedic Surgery<br>Brouwer, Reinoud; Erasmus Medical Center Rotterdam / Martini General hospital Groningen, Orthopaedic surgery<br>Veeger, Nic; Universitair Medisch Centrum Groningen, Department of Epidemiology<br>van Hulst, Marinus; Martini Hospital, Clinical Pharmacy; UMCG, Department of Health Sciences
Keywords:	Thromboembolism < CARDIOLOGY, Anticoagulation < HAEMATOLOGY, Knee < ORTHOPAEDIC & TRAUMA SURGERY, Bleeding disorders & coagulopathies < HAEMATOLOGY

SCHOLARONE™
Manuscripts

I, the Submitting Author has the right to grant and does grant on behalf of all authors of the Work (as defined in the below author licence), an exclusive licence and/or a non-exclusive licence for contributions from authors who are: i) UK Crown employees; ii) where BMJ has agreed a CC-BY licence shall apply, and/or iii) in accordance with the terms applicable for US Federal Government officers or employees acting as part of their official duties; on a worldwide, perpetual, irrevocable, royalty-free basis to BMJ Publishing Group Ltd ("BMJ") its licensees and where the relevant Journal is co-owned by BMJ to the co-owners of the Journal, to publish the Work in this journal and any other BMJ products and to exploit all rights, as set out in our licence.

The Submitting Author accepts and understands that any supply made under these terms is made by BMJ to the Submitting Author unless you are acting as an employee on behalf of your employer or a postgraduate student of an affiliated institution which is paying any applicable article publishing charge ("APC") for Open Access articles. Where the Submitting Author wishes to make the Work available on an Open Access basis (and intends to pay the relevant APC), the terms of reuse of such Open Access shall be governed by a Creative Commons licence – details of these licences and which Creative Commons licence will apply to this Work are set out in our licence referred to above.

Other than as permitted in any relevant BMJ Author's Self Archiving Policies, I confirm this Work has not been accepted for publication elsewhere, is not being considered for publication elsewhere and does not duplicate material already published. I confirm all authors consent to publication of this Work and authorise the granting of this licence.

For peer review only

Bleeding complications of thromboprophylaxis with dabigatran, nadroparin or rivaroxaban for six weeks after total knee arthroplasty surgery – a randomised pilot study

Lucia van der Veen[1,2], Marijn Segers[1] Jos JAM van Raay[3], Carina L E Gerritsma-Bleeker[3], Reinoud W. Brouwer[3], Nic JGM Veeger[4], Marinus van Hulst[1,5]

1. Department of Clinical Pharmacy and Toxicology, Martini Hospital (Groningen, The Netherlands)
2. Department of Clinical Pharmacy, Ommelander Hospital Groningen (Winschoten, The Netherlands)
3. Department of Orthopaedic Surgery, Martini Hospital (Groningen, The Netherlands)
4. Department of Epidemiology, University of Groningen, University Medical Center Groningen, Groningen, The Netherlands
5. Department of Health Sciences, University of Groningen, University Medical Center Groningen (Groningen, The Netherlands)

Author responsible for correspondence: M. van Hulst PhD, PharmD

Martini Hospital
Department of Clinical Pharmacy and Toxicology
Van Swietenplein1
9728 NT Groningen, The Netherlands

UMCG
Department of Health Sciences
University of Groningen
University Medical Center Groningen
Groningen, the Netherlands

Tel.: ++31-50-5247382
e-mail: m.van.hulst@rug.nl

Trial registration number: ClinicalTrails.gov: NCT01431456

For peer review only

Abstract

Objectives: For the non-vitamin-K oral anticoagulants, data on bleeding when used for 42 days as thromboprophylaxis after TKA is scarce. This pilot study assessed feasibility of a multicentre randomised clinical trial to evaluate major and clinically relevant non-major bleeding during 42-day use of dabigatran, nadroparin and rivaroxaban after TKA.

Patients and Methods: In 70 weeks, 198 TKA patients were screened for eligibility in the Martini Hospital (Groningen, The Netherlands). Patients were randomly assigned to dabigatran (n=45), nadroparin (n=45), or rivaroxaban (n=48). The primary outcome was the combined end-point of major bleeding and clinically relevant non-major bleeding. Secondary endpoints of this study were the occurrence of clinical VTE (PE or DVT), compliance, duration of hospital stay, re-hospitalisation, adverse events and Knee injury and Osteoarthritis Outcome Score (KOOS).

Results: The primary outcome was observed in 33.3% (95%CI:20.0%–49.0%), 24.4% (95%CI:12.9%–39.5%) and 27.1% (95%CI:15.3%–41.8%) of patients who received dabigatran, nadroparin, or rivaroxaban, respectively (p=0.67). Major bleeding was found in two patients who received nadroparin (p=0.21). Clinically relevant non-major bleeding was observed in 33.3% (95%CI:20.0%–49.0%), 22.2% (95%CI:11.2%–37.1%), 27.1% (95%CI:15.3%-41.8%) for dabigatran, nadroparin and rivaroxaban, respectively (p=0.51). Wound hematoma was the most observed bleeding event. VTE was found in one patient who received dabigatran (p=0.65). The pre- and post-surgery KOOS questionnaires were available for 32 (71%), 35 (77%) and 35 (73%) patients for dabigatran, nadroparin and rivaroxaban, respectively. KOOS was highly variable, and no significant difference between treatment groups in mean improvement was observed.

Conclusions: A multicentre clinical trial may be feasible. However, investments will be substantial. No differences in major and non-major clinically relevant bleeding events were found between dabigatran, nadroparin and rivaroxaban during 42 days after TKA. KOOS may not be suitable to detect functional loss due to bleeding.

Article Summary

Strengths and limitations of this study

For peer review only

Strengths

- Direct comparison of dabigatran, rivaroxaban and nadroparin as thromboprophylaxis after total knee arthroplasty
- Evaluation of extended thromboprophylaxis for 42 days

Limitations

- Pilot-study, hence limited number of included patients
- Although aimed to enhance external validity, a large number of patients had to be excluded

For peer review only

Keywords: thrombosis, orthopaedic surgery, DOAC, NOAC, LMWH.

Word count

3,037

Introduction

After total knee arthroplasty (TKA) surgery, patients are at risk to develop venous thromboembolism (VTE). Without thromboprophylaxis, (venographic) DVT can be found in 40 – 80% of patients.[1,2] The risk of non-fatal and fatal PE for TKA surgery patients without thromboprophylaxis is 1.8% – 7.0% and 0.2% – 0.7%, respectively.[3] Hence, thromboprophylaxis is indicated for all patients undergoing TKA. Low molecular weight heparins (LMWHs) have become a gold standard in preventing VTE. Unfortunately, these anticoagulants have disadvantages such as, for example: a subcutaneous route of administration and risk of developing heparin-induced thrombocytopenia. These disadvantages can lead to low adherence of orthopaedic surgeons to the thromboprophylaxis guidelines and hamper compliance by patients.[4] During the last decades, oral anticoagulants were developed. Dabigatran, rivaroxaban, apixaban and edoxaban are four available non-vitamin-K oral anticoagulants (NOAC).

In clinical trials as well as in a real-world data study rivaroxaban was superior in preventing VTE compared to enoxaparin in TKA surgery patients.[5-7] However, compared to enoxaparin, rivaroxaban was associated with a slightly increased risk of postoperative bleeding and wound complications.[8] Clinical trials and subsequent meta-analyses showed that VTE risk and bleeding risk are similar for dabigatran compared to LMWH in TKA.[8-11]

The external validity of the rivaroxaban and dabigatran clinical trials may be limited for Dutch TKA patients. Next to that enoxaparin was used in the clinical trials, whereas in the Netherlands nadroparin is predominantly used, also the duration is extended in the Netherlands.[12] In the market authorisation for dabigatran and rivaroxaban the duration of VTE prevention is limited to 10 – 14 days. In our study, thromboprophylaxis was offered to patients for a period of 42 days after surgery in agreement with Dutch and regional guidelines.[12],[13] Moreover, clinical trials have demonstrated that a more prolonged prophylaxis with LMWH after hospital discharge significantly reduces the incidence of venographically detected DVT and PE after arthroplasty.[14,15]

For peer review only

The objective of our study was to compare the 42 day use of dabigatran, nadroparin and rivaroxaban on safety after TKA in a randomised open label pilot study by assessing the risk of major and clinically relevant non-major bleeding using a standardized model of bleeding definitions,[16] in order to obtain insight into the design of a multi-centre study investigating the safety of the new oral anticoagulants for prevention of venous thromboembolism after TKA in the Dutch setting. The rationale of our study was previously published.[17]

For peer review only

Methods

Study design

This study was an open label randomized pilot trial with a three-arm design, conducted at the Martini Hospital in Groningen, The Netherlands. This study was approved by the Medical Ethical committee "Regionale Toetsingscommissie Patiëntgebonden Onderzoek" (RTPO) in Leeuwarden, The Netherlands. This clinical trial was registered under NCT01431456 at clinicaltrials.gov. At the start of the study, an independent hospital pharmacist assigned the three treatment groups to a consecutive series of numbers, using a computer-generated randomised list. Before surgery, patients were included and randomly assigned to one of the three treatment groups (dabigatran, rivaroxaban or nadroparin). The allocation concealment was reached by using the sequentially numbered opaque sealed envelopes (SNOSE) method.[18] On enrolment, each patient was assigned to the next consecutive treatment number and the corresponding study medication was dispensed in an open-label fashion. Patients or the public were not involved in the design, or reporting, or dissemination plans of our research as this study is a pilot study. Patients were involved in the conduct of the trial by sharing experiences during the visits.

Study population

The target population consisted of TKA patients. Patients ≥ 18 years and weighing more than 40 kg who were scheduled for primary elective TKA and had provided signed informed consent were eligible for the study. Exclusion criteria included: a known inherited or acquired clinically significant active high risk of bleeding or bleeding disorder; major surgery, trauma, uncontrolled severe arterial hypertension, or myocardial infarction within the last three months; history of acute intracranial disease or haemorrhagic stroke; gastrointestinal or urogenital bleeding or ulcer disease within the last six months; cirrhotic patients with moderate hepatic impairment (aspartate or alanine aminotransferase levels higher than twice the upper limit of the normal range within the last month); severe renal insufficiency (creatinine clearance < 30 mL/min); other indication for treatment with anticoagulants; active malignant disease; pregnancy or breastfeeding. Patients received standard orthopaedic care

For peer review only

and physiotherapy according to the local standardised protocol. During the enrolment of the trial, fast-track knee replacement surgery was implemented.[19,20]

Treatment regimens

Patients were assigned to oral dabigatran etexilate 150 mg or 220 mg (two capsules, 75 mg or 110 mg) once daily, subcutaneous nadroparin 2850 IE = 0.3 ml once daily or 10 mg of oral rivaroxaban once daily. The first dose of dabigatran was one-half of subsequent doses (one capsule, 75 mg or 110 mg). Treatment was continued for a total of 42 days after TKA.

Outcome measures

The primary endpoint of this study was a major bleeding event and or clinically relevant non-major bleeding event within the 42-days treatment period. Any bleeding event that occurred during the 42-days treatment period, perceived by patient, researcher, nurse, orthopaedic surgeon or other health worker were registered and evaluated. A blinded independent expert adjudication committee classified all bleeding events according to international guidelines.[16] A major bleeding was defined as fatal bleeding, clinically overt bleeding associated with a decrease in the haemoglobin level of more than 20 g/l compared with the pre-randomisation level, clinically overt bleeding leading to transfusion of ≥2 units of whole blood or packed cells, critical bleeding (intracerebral, intraocular, intraspinal, pericardial or retroperitoneal), bleeding warranting treatment cessation, bleeding located at the surgical site and leading to re-operation or to any unusual medical intervention or procedure for relief (eg, draining or puncture of a haematoma at the surgical site, transfer to an ICU or emergency room). Clinically relevant non-major bleeding was defined as spontaneous skin haematoma >25cm^2, wound haematoma >100cm^2, spontaneous nose bleeding or gingival bleeding lasting longer than 5 min, spontaneous rectal bleeding creating more than a spot on toilet paper, macroscopic haematuria either spontaneous or, if associated with an intervention, lasting longer than 24 h, other bleeding events considered clinically relevant by the investigator not qualifying as a major bleeding.

For peer review only

Secondary endpoints of this study were the occurrence of clinical VTE (PE or DVT), compliance, duration of hospital stay, re-hospitalisation, adverse events and Knee injury and Osteoarthritis Outcome Score (KOOS).[21]

The duration of hospital stay and re-hospitalisation were retrieved from electronic medical records.

Compliance was measured by counting the number of doses left over after 42 days of treatment. In our study, patients were deemed compliant if patients didn't miss a single dose.

Adverse events were included in the analysis if the relationship with the study drug was possible, probable or highly probable.

The KOOS (www.koos.nu) is a 42-item self-administered questionnaire that includes five dimensions: pain, disease-related symptoms, activities of daily living function (ADL), sport and recreation function, and knee-related quality of life measured using a Likert scale (0 – 4 scale).[21] The KOOS was administered pre-surgery and six weeks after TKA. A patient with an improvement of ten points or more on a KOOS dimension after TKA was defined as responder.

Statistical analysis

The absolute bleeding risk was estimated as the percentage of patients with a bleed for each treatment group, with the associated 95% confidence intervals. Confidence intervals were derived using the (exact) Clopper-Pearson method. The risk of thromboembolic events was assessed using the same methodology. The analysis was based on the per-protocol population, i.e. all fully compliant patients that were evaluated 42 days after surgery. One-way analysis of variance (ANOVA) was used for analyses of continuous variables between multiple groups. If normality assumption was violated (Shapiro-Wilk test for normality), the non-parametric Kruskal Wallis test was used. Distribution of nominal variables for different groups was tested for statistical significance using the exact Chi-squared test. All statistical analyses were performed using IBM Statistics SPSS 25.0.0. Due to the explorative nature of this pilot study, all *p*-values will be interpreted as explorative rather than confirmative.[22]

Results

For peer review only

Between July 2012 and November 2013, a total of 198 patients were assessed for eligibility of which 148 patients were randomised to treatment. In total 138 patients were included in the final analysis, see flow diagram (Figure 1). The three treatment groups were well balanced in terms of baseline demographics (Table 1). Of all patients, 63% were woman, with a mean age of 66 years and a mean weight of 89 kg. The mean time from end of surgery to the first study drug intake was 8 hours and 48 minutes. In total, 111 (80%) patients had fast-track TKA. Compared to patients in the other treatment groups, a larger share of patients in the dabigatran group had fast-track TKA. Thirty-three (24%) patients received a patella prosthesis during TKA. Compared to the nadroparin group, a larger share of patients in the rivaroxaban group and smaller share of patients in the dabigatran group received a new patella. Pre-surgery KOOS was well balanced between the three treatment groups.

Primary endpoints

The combined endpoint of major and clinically relevant non-major bleeding was observed in 15 of 45 (33.3%; 95% CI 20.0% – 49.0%) patients who received dabigatran, 11 of 45 (24.4%; 95% CI 12.9% – 39.5%) patients who received nadroparin and 13 of 48 (27.1%; 95% CI 15.3% – 41.8%) patients who received rivaroxaban. There was no significant difference in these bleeding events between dabigatran, rivaroxaban and nadroparin (p = 0.67), see Table 2.

Major bleeding events occurred in none of 45 (0%; 95% CI 0% – 7.9%) patients in the dabigatran group, two of 45 (4.4%; 95% CI 0.5% – 15.1%) patients in the nadroparin group and none of 48 (0%; 95% CI 0% – 7.4%) patients in the rivaroxaban group. One of these events was a clinically overt bleeding from the wound leading to transfusion of 2 or more units of whole blood or packed cells and one was adjudicated as a subdural hematoma caused by a fall. There was no significant difference in major bleeding events between dabigatran, nadroparin and rivaroxaban (p = 0.21).

Clinically relevant non-major bleeding events were observed in 15 patients who received dabigatran (33.3%; 95% CI 20.0% – 49.0%), 10 patients who received nadroparine (22.2%; 95% CI 11.2% – 37.1%),) and 13 patients who received rivaroxaban (27.1%; 95% CI 15.3% – 41.8%). Most of these clinically relevant non-major bleeding events were wound hematomas.

For peer review only

There was no significant difference in clinically relevant non-major bleeding events between dabigatran, rivaroxaban and nadroparin (p = 0.51). The last major or clinically relevant non-major bleeding event was observed a median seven, seven and six days post-operatively, for dabigatran, nadroparin and rivaroxaban, respectively (p = 0.32). In the period between 14 and 42 days after surgery, one major or clinically relevant non-major bleeding event was observed in all treatment groups (p = 1.0).

Secondary endpoints

The secondary endpoints of this study were: occurrence of clinical VTE, hospital stay, re-admission, adverse events, compliance and KOOS, see Table 2 and 3. Clinical VTE occurred in one of 45 patients receiving dabigatran and in none receiving rivaroxaban or nadroparin. Unfortunately, this patient with VTE developed PE and died. The median duration of hospital stay was three, three and four days for dabigatran, nadroparine and rivaroxaban, respectively (p = 0.098). Re-admission within three months post-surgery was not significantly different between treatment groups (p = 0.77). The number of patients re-admitted within three months was two (4.4%), four, (8.9%) and three (6.3%) patients, for dabigatran, nadroparin and rivaroxaban, respectively. Adverse events were reported in nine (20%), two (4.4%) and five (10.4%) patients for dabigatran, nadroparin and rivaroxaban, respectively (p = 0.072). Five patients in the dabigatran group (11.1%) experienced gastro-intestinal adverse effects such as abdominal pain, diarrhoea and/or indigestion. In the rivaroxaban group, gastro-intestinal adverse effects were found in two patients (4.4%).

In the dabigatran group, 27 of 44 patients (61.4%) were compliant versus 25 of 44 patients (56.8%) in the nadroparine group and 25 of 48 patients in the rivaroxaban group (52.1%). Compliance was not significantly different between treatment groups (p = 0.66). Also, the number of missed days of treatment was not significantly different between treatment groups (p = 0.73). In the period between the 42-day follow-up and three months post-surgery, we observed no additional bleeding and VTE events.

For peer review only

The pre- and post-surgery KOOS questionnaires were available for 32 (71%), 35 (77%) and 35 (73%) patients for dabigatran, nadroparin and rivaroxaban, respectively (Table 3). No significant difference between treatment groups in mean improvement was observed for the KOOS dimensions pain, symptoms, ADL, sport and recreation and quality of life. Also, the proportion of responders, patients with a ten point or more improvement on a KOOS dimension, was not significantly different between dabigatran, nadroparin and rivaroxaban. The standard deviation in KOOS was mostly equal or even higher than the mean.

Discussion

After knee replacement, when bleeding in the replaced joint can have serious consequences, the balance between inducing bleeding and preventing VTE is pivotal in thromboprophylaxis. In this study, we compared bleeding complications of dabigatran, rivaroxaban and nadroparin when used to prevent VTE after TKA surgery. This exploratory study shows that there was no significant difference in major and clinically relevant bleeding between dabigatran, rivaroxaban and nadroparin. The bleeding risk we found is higher than observed in previous knee replacement studies. In such studies a risk of non-major bleeding events of between 2.6% and 6.8% was observed.[5,6,23] In our study, the risk of bleeding events is higher mainly due to the risk of wound haematomas > 100 cm^2. A possible explanation for the difference in occurrence of clinically relevant non-major bleeding events is our strict adherence to EMA bleeding definitions.[16 24] For instance, in the RECORD 3 and RECORD 4 rivaroxaban trials, surgical site and wound haematomas were not reported.[5,6,8,24]

The market authorisation for the prevention of venous thromboembolism after total knee arthroplasty for dabigatran and rivaroxaban is 10-14 days. In our study, patients received thromboprophylaxis for 42 days. Most bleeding events occurred during the first 14 days post-surgery. Notably, bleeding risks after the first 14 days use of dabigatran, nadroparin and rivaroxaban were low and not different between treatment groups. Based on our pilot-study and a meta-analysis of dabigatran and rivaroxaban in TKA, it is expected that large numbers of patients have to be included in a clinical trial to detect a clinically relevant difference in major and clinically relevant non-major bleeding between NOACs and LMWHs when used for

42 days.[8] Therefore, costs of conducting a multicentre clinical trial based on the evaluated protocol and a formal power calculation are expected to be very high.

As expected, the number of symptomatic VTE cases was very low. Unfortunately, one case of PE occurred in the dabigatran group, leading to the death of the patient.

It is generally assumed that patients would prefer the oral route to subcutaneous injections. However, it is unknown if the route of administration has an impact on patient compliance. In our study, we found no clear indication for a difference in compliance between rivaroxaban, nadroparin and dabigatran.

Although we found no differences in the number of patients with adverse reactions, the number of patients with gastro-intestinal adverse reaction in the dabigatran group appeared to be higher than in the other treatment groups. Dabigatran is also in other clinical trials associated with gastro-intestinal adverse effects.[25] Between dabigatran, nadroparin and rivaroxaban no difference in patient reported outcomes as assessed with the KOOS was found. The hypothesis was that joint haemorrhages could impair subjective improvement after TKA as assessed by KOOS. Our pilot study shows that it is feasible to assess KOOS in a study evaluating bleeding complications of thromboprophylaxis. However, variability in KOOS is probably too high to detect differences in KOOS in a future multicentre clinical trial.

The results of this pilot study should be interpreted with caution due to some limitations. Firstly, as a pilot-study, the size of the study population is limited. Although our aim was to conduct a pilot study to design a multicentre study with an improved external validity compared to available randomised clinical trials, we had to exclude a large number of patients some of who will receive thromboprophylaxis with NOACs in regular clinical practice. Additionally, we had to exclude patients with a high risk of bleeding who may receive thromboprophylaxis with NOACs in regular clinical practice.

Conclusion

For peer review only

A multicentre clinical trial may be feasible based on the recruitment speed and satisfaction with the trial protocol. No differences in major and non-major clinically relevant bleeding events were found in a pilot-study of the head-to-head comparison of dabigatran, nadroparin and rivaroxaban as 42-day thromboprophylaxis in TKA. After the first 14 days use of dabigatran, nadroparin or rivaroxaban, bleeding risks were low and not different. This finding implies that conducting a multicentre trial will require substantial investment to achieve an adequate number of evaluated patients. Furthermore, due to the high variability in the KOOS, this outcome score may not be suitable to detect functional loss due to bleeding in a future clinical trial.

Acknowledgements

The adjudication committee (mrs A. van der Aart, hospital pharmacist; mrs A. van der Velden, internist-haematologist / oncologist and dr. R.W. Brouwer, orthopaedic surgeon) is thanked for the careful assessment of bleeding events. Dr Brouwer joint the writing committee after adjudication of bleeding events. Mrs Roya Sorghabi is thanked for clinical trial management and data acquisition. Nurses and doctors of the orthopaedic surgery department are thanked for screening patients for bleeding events.

Author Contributions

LV, MH, CG and JR conceived the idea of the study and were responsible for the design of the study. NV, LV and MH performed the statistical analysis for the study. The initial draft of the manuscript was prepared by LV, MS, MH, JR, RB and NV and then circulated repeatedly among all authors for critical revision. LV and MS were responsible for the acquisition of the data and MH and NV contributed to the interpretation of the results. LV was the coordinator of the study and lead writer. All authors helped plan the study, evolve analysis plans and critically revise successive drafts of the manuscript.

Funding

This research received no specific grant from any funding agency in the public, commercial or not-for-profit sectors.

For peer review only

Competing interests

MH reports grants from Bayer and personal fees from Boehringer Ingelheim during the conduct of the study. Other authors have nothing to report.

Data sharing

Data can made be available upon request to the corresponding author.

For peer review only

Table 1. Patient characteristics

	Dabigatran n = 45	Nadroparine n = 45	Rivaroxaban n = 48	Total n = 138
Female n (%)	26 (58%)	31 (69%)	30 (63%)	87 (63%)
Age (year) [±SD]	66±7.9	67±10	65±7.5	66±8.7
Weight (kg) [±SD]	88±12	89±18	90±21	89±17
Fast-track surgery (%)	41 (91%)	35 (78%)	35 (73%)	111 (80%)
Received new patella (%)	7 (16%)	10 (22%)	16 (33%)	33 (24%)
History of VTE (%)	1 (2.2%)	1 (2.2%)	2 (4.2%)	4 (2.9%)
Time to first dose (h) [±SD]	8.6±2.3	9.0±2.2	8.8±2.1	8.8±2.2
Pre-surgery KOOS [±SD]				
- pain {n}	47±15 {32}	48±20 {35}	51±21 {35}	49±19 {102}
- symptoms {n}	55±16 {32}	49±18 {35}	50±18 {35}	51±17 {102}
- ADL {n}	52±15 {32}	48±19 {35}	56±19 {35}	52±18 {102}
- sport and recreation {n}	23±25 {29}	24±28 {31}	22±22 {29}	23±25 {89}
- quality of life {n}	30±12 {31}	30±16 {35}	30±15 {35}	30±14 {101}

ADL: activities of daily living function; KOOS: Knee injury and Osteoarthritis Outcome Score; SD: standard deviation; VTE: venous thromboembolism.

For peer review only

Table 2. Primary and secondary outcomes of thromboprophylaxis with dabigatran, nadroparin or rivaroxaban after total knee arthroplasty

	Dabigatran n = 45		Nadroparin n = 45		Rivaroxaban n = 48		*p*-value
	n	*%*	*n*	*%*	*n*	*%*	
Primary outcome							
Major and clinically relevant non-major bleeding	15	33.3	11	24.4	13	27.1	$p = 0.67$
Major bleedings	0	0	2	4.4	0	0	$p = 0.21$
Fatal bleeding	0	0	0	0	0	0	
Clinically overt bleeding, Hb decrease>20 g/l	0	0	0	0	0	0	
Clinically overt bleeding, transfusion	0	0	1	2.2	0	0	$p = 0.65$
Critical bleeding	0	0	1*	2.2	0	0	$p = 0.65$
Bleeding warranting treatment cessation	0	0	1*	2.2	0	0	$p = 0.65$
Bleeding located at surgical site	0	0	0	0	0	0	
Clinically relevant non-major bleeding	15	33.3	10	22.2	13	27.1	$p = 0.51$
Spontaneous skin haematoma >25 cm2	1	2.2	0	0	0	0	$p = 0.65$
Wound haematoma >100 cm2	14	31.1	9	20.0	12	25.0	$p = 0.49$
Spontaneous nose bleeding > 5 min	0	0	1	2.2	0	0	$p = 0.65$
Spontaneous rectal bleeding > 1 spot	0	0	0	0	0	0	
Macroscopic haematuria > 24 h	0	0	0	0	1	2.1	$p = 1.0$
Other bleeding event (not major)	0	0	0	0	0	0	
Last primary outcome (median days after surgery) [range]	7 [1 – 21]		7 [2 – 26]		6 [2 – 16]		$p = 0.32$
Patients with primary outcome more than 14 days after surgery (%)	1	2.2	1	2.2%	1	2.1	$p = 1.0$
Secondary outcome							
VTE	1*	2.2	0	0	0	0	$p = 0.65$
Death due to VTE	1*		0		0		
DVT	0		0		0		
PE	1*		0		0		
Hospital stay (median, days) [range]	3 [2 – 9]		3 [2 – 10]		4 [2 – 28]		$p = 0.098$
Re-admission within three months (%)	2 (4.4%)		4 (8.9%)		3 (6.3%)		$p = 0.77$
Patients with adverse reactions (%)	9 (20.0%)		2 (4.4%)		5 (10.4%)		$p = 0.072$
Number of patient compliant (%)	27 of 44 (61.4%)†		25 of 44 (56.8%)†		25 (52.1%)		$p = 0.66$
Days of missed dosages (median, days) [range]	0 [0 – 7]; n=42		0 [0 – 23]; n=40		0 [0 – 4]; n=40		$p = 0.73$

VTE: venous thromboembolism; PE: pulmonary embolism; DVT: deep venous thrombosis; SD: standard deviation; *identical patient; † one missing value.

For peer review only

Table 3. Knee injury and Osteoarthritis Outcome Score (KOOS) for patients with dabigatran, nadroparin or rivaroxaban thromboprophylaxis after total knee arthroplasty. Difference between six weeks post-surgery and pre-surgery KOOS and proportion of patients with 10 points or more improvement in KOOS between six weeks post-surgery and pre-surgery.

	Dabigatran	Nadroparin	Rivaroxaban	*p*-value
Pain	n = 32	n = 35	n = 35	
Difference six weeks post-surgery and pre-surgery (mean±SD)	25±26	19±22	22±23	p=0.51
Patients with ≥10 points improvement post- vs pre-surgery (n; %)	22; 69%	26; 74%	27; 77%	p=0.78
Symptoms	n = 32	n = 35	n = 35	
Difference six weeks post-surgery and pre-surgery (mean±SD)	12±20	14±20	16±19	p=0.63
Patients with ≥10 points improvement post- vs pre-surgery (n, %)	17; 53%	20; 57%	22; 63%	p=0.72
ADL	n = 32	n = 35	n = 35	
Difference six weeks post-surgery and pre-surgery (mean±SD)	25±20	23±21	21±17	p=0.75
Patients with ≥10 points improvement post- vs pre-surgery (n, %)	23; 72%	25; 71%	27; 77%	p=0.88
Sport and recreation	n = 29	n = 31	n = 29	
Difference six weeks post-surgery and pre-surgery (mean±SD)	24±38	25±41	28±33	p=0.89
Patients with ≥10 points improvement post- vs pre-surgery (n, %)	17; 59%	21; 68%	23;79%	p=0.24
Quality of Life	n = 31	n = 35	n = 35	
Difference six weeks post-surgery and pre-surgery (mean±SD)	26±27	18±20	22±21	p=0.39
Patients with ≥10 points improvement post- vs pre-surgery (n, %)	24;77%	23;66%	23; 66%	p=0.55

ADL: activities of daily living function; SD: standard deviation.

For peer review only - http://bmjopen.bmj.com/site/about/guidelines.xhtml

Figure 1. Flow diagram. AF: atrial fibrillation; ALT: alanine aminotransferase; AST: aspartate aminotransferase.

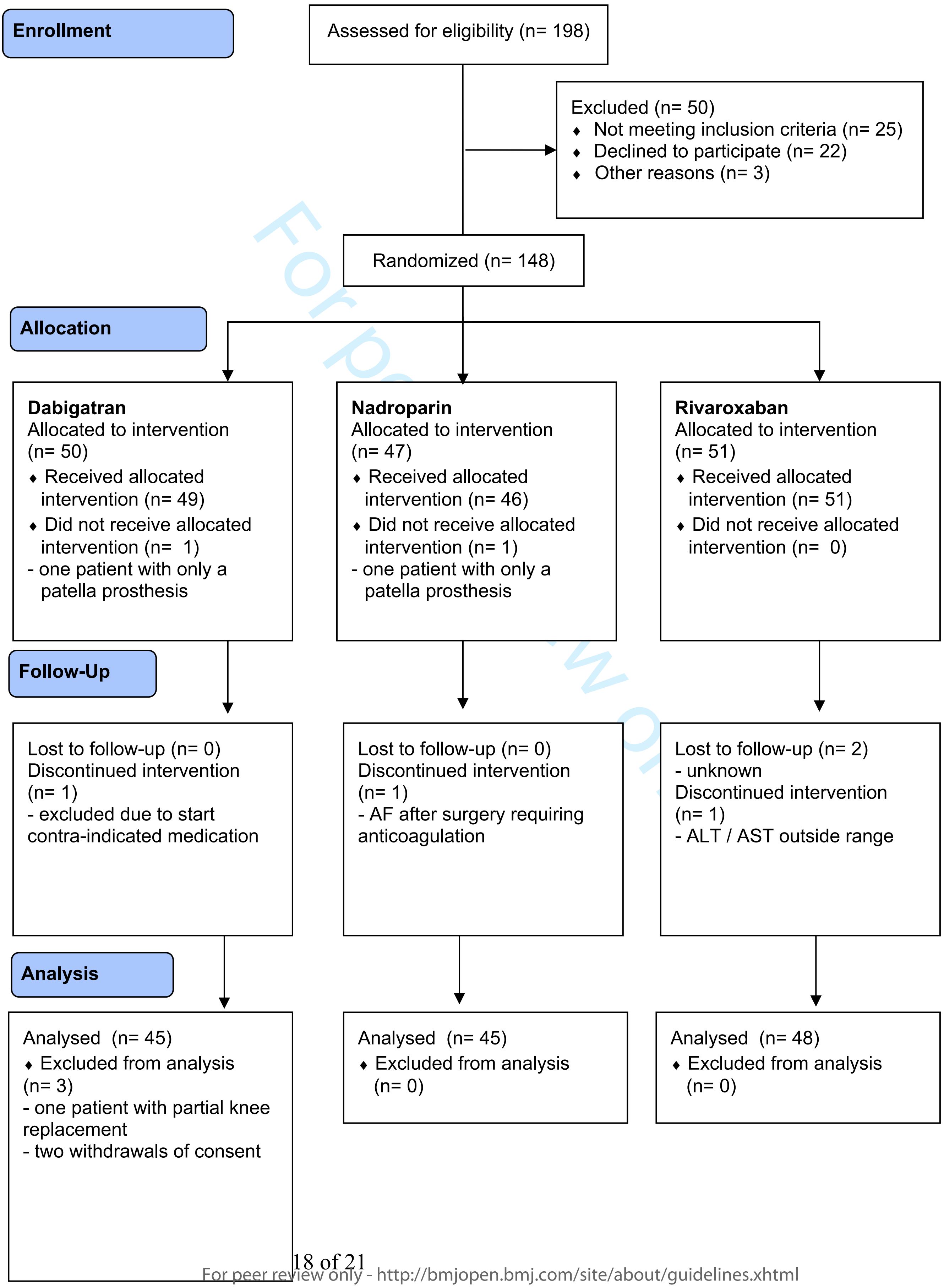

For peer review only - http://bmjopen.bmj.com/site/about/guidelines.xhtml

For peer review only

For peer review only - http://bmjopen.bmj.com/site/about/guidelines.xhtml

Reference list

1. Warwick D, Friedman RJ, Agnelli G, et al. Insufficient duration of venous thromboembolism prophylaxis after total hip or knee replacement when compared with the time course of thromboembolic events: findings from the Global Orthopaedic Registry. *The Journal of bone and joint surgery British volume* 2007; **89**(6): 799-807.
2. White RH. The epidemiology of venous thromboembolism. *Circulation* 2003; **107**(23 Suppl 1): I4-8.
3. Falck-Ytter Y, Francis CW, Johanson NA, et al. Prevention of VTE in orthopedic surgery patients: Antithrombotic Therapy and Prevention of Thrombosis, 9th ed: American College of Chest Physicians Evidence-Based Clinical Practice Guidelines. *Chest* 2012; **141**(2 Suppl): e278S-325S.
4. Rachidi S, Aldin ES, Greenberg C, Sachs B, Streiff M, Zeidan AM. The use of novel oral anticoagulants for thromboprophylaxis after elective major orthopedic surgery. *Expert review of hematology* 2013; **6**(6): 677-95.
5. Lassen MR, Ageno W, Borris LC, et al. Rivaroxaban versus enoxaparin for thromboprophylaxis after total knee arthroplasty. *The New England journal of medicine* 2008; **358**(26): 2776-86.
6. Turpie AG, Lassen MR, Davidson BL, et al. Rivaroxaban versus enoxaparin for thromboprophylaxis after total knee arthroplasty (RECORD4): a randomised trial. *Lancet* 2009; **373**(9676): 1673-80.
7. Turpie AG, Haas S, Kreutz R, et al. A non-interventional comparison of rivaroxaban with standard of care for thromboprophylaxis after major orthopaedic surgery in 17,701 patients with propensity score adjustment. *Thrombosis and haemostasis* 2014; **111**(1): 94-102.
8. Gomez-Outes A, Terleira-Fernandez AI, Suarez-Gea ML, Vargas-Castrillon E. Dabigatran, rivaroxaban, or apixaban versus enoxaparin for thromboprophylaxis after total hip or knee replacement: systematic review, meta-analysis, and indirect treatment comparisons. *BMJ* 2012; **344**: e3675.
9. Huisman MV, Quinlan DJ, Dahl OE, Schulman S. Enoxaparin versus dabigatran or rivaroxaban for thromboprophylaxis after hip or knee arthroplasty: Results of separate pooled analyses of phase III multicenter randomized trials. *Circulation Cardiovascular quality and outcomes* 2010; **3**(6): 652-60.
10. Friedman RJ, Dahl OE, Rosencher N, et al. Dabigatran versus enoxaparin for prevention of venous thromboembolism after hip or knee arthroplasty: a pooled analysis of three trials. *Thrombosis research* 2010; **126**(3): 175-82.
11. Venker BT, Ganti BR, Lin H, Lee ED, Nunley RM, Gage BF. Safety and Efficacy of New Anticoagulants for the Prevention of Venous Thromboembolism After Hip and Knee Arthroplasty: A Meta-Analysis. *The Journal of arthroplasty* 2017; **32**(2): 645-52.
12. Vereniging NI. Keuze en duur van tromboseprofylaxe bij grote orthopedische en traumatologische ingrepen. https://richtlijnendatabase.nl/richtlijn/antitrombotisch_beleid/preventie_vte/keuze_en_duur_profylaxe_grote_ingrepen.html#verantwoording (accessed 13-02-2018).
13. Ettema HB, Mulder MC, Nurmohamed MT, Buller HR, Verheyen CC. Dutch orthopedic thromboprophylaxis: a 5-year follow-up survey. *Acta orthopaedica* 2009; **80**(1): 109-12.
14. White RH, Romano PS, Zhou H, Rodrigo J, Bargar W. Incidence and time course of thromboembolic outcomes following total hip or knee arthroplasty. *Archives of internal medicine* 1998; **158**(14): 1525-31.

For peer review only - http://bmjopen.bmj.com/site/about/guidelines.xhtml

15. Bergqvist D, Benoni G, Bjorgell O, et al. Low-molecular-weight heparin (enoxaparin) as prophylaxis against venous thromboembolism after total hip replacement. *The New England journal of medicine* 1996; **335**(10): 696-700.
16. (EMA) EMA. Guideline on clinical investigation of medicinal products for the prophylaxis of high intra- and postoperative venous thromboembolic risk. *CPMP/EWP/707/98* 2007; **15-11-2007**.
17. Veen L, van Raay JJ, Gerritsma-Bleeker CL, Veeger NJ, Hulst M. Direct treatment comparison of DAbigatran and RIvaroxaban versus NAdroparin in the prevention of venous thromboembolism after total knee arthroplasty surgery: design of a randomised pilot study (DARINA). *BMJ open* 2013; **3**(1).
18. Doig GS, Simpson F. Randomization and allocation concealment: a practical guide for researchers. *Journal of critical care* 2005; **20**(2): 187-91; discussion 91-3.
19. Husted H, Jensen CM, Solgaard S, Kehlet H. Reduced length of stay following hip and knee arthroplasty in Denmark 2000-2009: from research to implementation. *Archives of orthopaedic and trauma surgery* 2012; **132**(1): 101-4.
20. Van Egmond JC, Verburg H, Mathijssen NM. The first 6 weeks of recovery after total knee arthroplasty with fast track. *Acta orthopaedica* 2015; **86**(6): 708-13.
21. de Groot IB, Favejee MM, Reijman M, Verhaar JA, Terwee CB. The Dutch version of the Knee Injury and Osteoarthritis Outcome Score: a validation study. *Health and quality of life outcomes* 2008; **6**: 16.
22. Julious SA. Sample size of 12 per group rule of thumb for a pilot study. *Pharmaceutical Statistics* 2005; **4**(4): 5.
23. Eriksson BI, Dahl OE, Rosencher N, et al. Oral dabigatran etexilate vs. subcutaneous enoxaparin for the prevention of venous thromboembolism after total knee replacement: the RE-MODEL randomized trial. *Journal of thrombosis and haemostasis : JTH* 2007; **5**(11): 2178-85.
24. Dahl OE, Quinlan DJ, Bergqvist D, Eikelboom JW. A critical appraisal of bleeding events reported in venous thromboembolism prevention trials of patients undergoing hip and knee arthroplasty. *Journal of thrombosis and haemostasis : JTH* 2010; **8**(9): 1966-75.
25. Connolly SJ, Ezekowitz MD, Yusuf S, et al. Dabigatran versus warfarin in patients with atrial fibrillation. *The New England journal of medicine* 2009; **361**(12): 1139-51.

For peer review only

For peer review only - http://bmjopen.bmj.com/site/about/guidelines.xhtml

Reporting checklist for randomised trial.

Based on the CONSORT guidelines.

Instructions to authors

Complete this checklist by entering the page numbers from your manuscript where readers will find each of the items listed below.

Your article may not currently address all the items on the checklist. Please modify your text to include the missing information. If you are certain that an item does not apply, please write "n/a" and provide a short explanation.

Upload your completed checklist as an extra file when you submit to a journal.

In your methods section, say that you used the CONSORTreporting guidelines, and cite them as:

Schulz KF, Altman DG, Moher D, for the CONSORT Group. CONSORT 2010 Statement: updated guidelines for reporting parallel group randomised trials

		Reporting Item	Page Number
**Title and Abstract**			
Title	#1a	Identification as a randomized trial in the title.	Page 1
Abstract	#1b	Structured summary of trial design, methods, results, and conclusions	Page 2
**Introduction**			
Background and objectives	#2a	Scientific background and explanation of rationale	Page 4
Background and objectives	#2b	Specific objectives or hypothesis	Page 4
**Methods**			
Trial design	#3a	Description of trial design (such as parallel, factorial) including allocation ratio.	Page 6
Trial design	#3b	Important changes to methods after trial commencement (such as eligibility criteria), with reasons	Page 6

For peer review only

Participants	#4a	Eligibility criteria for participants	Page 6
Participants	#4b	Settings and locations where the data were collected	Page 6
Interventions	#5	The experimental and control interventions for each group with sufficient details to allow replication, including how and when they were actually administered	Page 6 – 7
Outcomes	#6a	Completely defined prespecified primary and secondary outcome measures, including how and when they were assessed	Page 7
Sample size	#7a	How sample size was determined.	Page 8
Sample size	#7b	When applicable, explanation of any interim analyses and stopping guidelines	Page 8
Randomization - Sequence generation	#8a	Method used to generate the random allocation sequence.	
Page 6			
Randomization - Sequence generation	#8b	Type of randomization; details of any restriction (such as blocking and block size)	
Page 6			
Randomization - Allocation concealment mechanism	#9	Mechanism used to implement the random allocation sequence (such as sequentially numbered containers), describing any steps taken to conceal the sequence until interventions were assigned	Page 6
Randomization - Implementation	#10	Who generated the allocation sequence, who enrolled participants, and who assigned participants to interventions	Page 6
Blinding	#11a	If done, who was blinded after assignment to interventions (for example, participants, care providers, those assessing outcomes) and how.	Page 6
Blinding	#11b	If relevant, description of the similarity of interventions	Page 6
Statistical methods	#12a	Statistical methods used to compare groups for primary and secondary outcomes	Page 8
Statistical methods	#12b	Methods for additional analyses, such as subgroup analyses and adjusted analyses	Page 8

For peer review only

Outcomes	#6b	Any changes to trial outcomes after the trial commenced, with reasons	Page 7
**Results**			
Participant flow diagram (strongly recommended)	#13a	For each group, the numbers of participants who were randomly assigned, received intended treatment, and were analysed for the primary outcome	Figure 1
Participant flow	#13b	For each group, losses and exclusions after randomization, together with reason	Figure 1
Recruitment	#14a	Dates defining the periods of recruitment and follow-up	Page 8
Recruitment	#14b	Why the trial ended or was stopped	n/a
Baseline data	#15	A table showing baseline demographic and clinical characteristics for each group	Page 15, table 1.
Numbers analysed	#16	For each group, number of participants (denominator) included in each analysis and whether the analysis was by original assigned groups	Figure 1
Outcomes and estimation	#17a	For each primary and secondary outcome, results for each group, and the estimated effect size and its precision (such as 95% confidence interval)	Page 9
Outcomes and estimation	#17b	For binary outcomes, presentation of both absolute and relative effect sizes is recommended	Page 9
Ancillary analyses	#18	Results of any other analyses performed, including subgroup analyses and adjusted analyses, distinguishing pre-specified from exploratory	Page 9 – 11
Harms	#19	All important harms or unintended effects in each group (For specific guidance see CONSORT for harms)	Page 10
**Discussion**			
Limitations	#20	Trial limitations, addressing sources of potential bias, imprecision, and, if relevant, multiplicity of analyses	Page 11
Interpretation	#22	Interpretation consistent with results, balancing benefits and harms, and considering other relevant evidence	Page 11
Registration	#23	Registration number and name of trial registry	Page 1

For peer review only

Other Information

Protocol	#24	Where the full trial protocol can be accessed, if available	Page 5 (ref 17) and Appendix
Funding	#25	Sources of funding and other support (such as supply of drugs), role of funders	Page 13

Notes:

- 15: Page 15, table 1.
- 18: Page 9 – 11
- 24: Page 5 (ref 17) and Appendix The CONSORT checklist is distributed under the terms of the Creative Commons Attribution License CC-BY. This checklist was completed on 24. April 2020 using https://www.goodreports.org/, a tool made by the EQUATOR Network in collaboration with Penelope.ai

For peer review only

BMJ Open

Bleeding complications of thromboprophylaxis with dabigatran, nadroparin or rivaroxaban for six weeks after total knee arthroplasty surgery – a randomised pilot study

Journal:	*BMJ Open*
Manuscript ID	bmjopen-2020-040336.R1
Article Type:	Original research
Date Submitted by the Author:	02-Dec-2020

Complete List of Authors:	van der Veen, Lucia; Martini Hospital, Clinical Pharmacy; Ommelander Hospital Groningen, Department of Clinical Pharmacy<br>Segers, Marijn; Martini Hospital, Department of Clinical Pharmacy and Toxicology<br>van Raay, Jos; Martini Hospital, Department of Orthopaedic Surgery<br>Gerritsma-Bleeker, Carina; Martini Hospital, Department of Orthopaedic Surgery<br>Brouwer, Reinoud; Martini Hospital, Orthopaedic surgery<br>Veeger, Nic; Universitair Medisch Centrum Groningen, Department of Epidemiology<br>van Hulst, Marinus; Martini Hospital, Clinical Pharmacy; UMCG, Department of Health Sciences

**Primary Subject Heading**:	Pharmacology and therapeutics
Secondary Subject Heading:	Surgery, Haematology (incl blood transfusion)
Keywords:	Thromboembolism < CARDIOLOGY, Anticoagulation < HAEMATOLOGY, Knee < ORTHOPAEDIC & TRAUMA SURGERY, Bleeding disorders & coagulopathies < HAEMATOLOGY

SCHOLARONE™
Manuscripts

I, the Submitting Author has the right to grant and does grant on behalf of all authors of the Work (as defined in the below author licence), an exclusive licence and/or a non-exclusive licence for contributions from authors who are: i) UK Crown employees; ii) where BMJ has agreed a CC-BY licence shall apply, and/or iii) in accordance with the terms applicable for US Federal Government officers or employees acting as part of their official duties; on a worldwide, perpetual, irrevocable, royalty-free basis to BMJ Publishing Group Ltd ("BMJ") its licensees and where the relevant Journal is co-owned by BMJ to the co-owners of the Journal, to publish the Work in this journal and any other BMJ products and to exploit all rights, as set out in our licence.

The Submitting Author accepts and understands that any supply made under these terms is made by BMJ to the Submitting Author unless you are acting as an employee on behalf of your employer or a postgraduate student of an affiliated institution which is paying any applicable article publishing charge ("APC") for Open Access articles. Where the Submitting Author wishes to make the Work available on an Open Access basis (and intends to pay the relevant APC), the terms of reuse of such Open Access shall be governed by a Creative Commons licence – details of these licences and which Creative Commons licence will apply to this Work are set out in our licence referred to above.

Other than as permitted in any relevant BMJ Author's Self Archiving Policies, I confirm this Work has not been accepted for publication elsewhere, is not being considered for publication elsewhere and does not duplicate material already published. I confirm all authors consent to publication of this Work and authorise the granting of this licence.

For peer review only

Bleeding complications of thromboprophylaxis with dabigatran, nadroparin or rivaroxaban for six weeks after total knee arthroplasty surgery – a randomised pilot study

Lucia van der Veen[1,2], Marijn Segers[1] Jos JAM van Raay[3], Carina L E Gerritsma-Bleeker[3], Reinoud W. Brouwer[3], Nic JGM Veeger[4], Marinus van Hulst[1,5]

1. Department of Clinical Pharmacy and Toxicology, Martini Hospital (Groningen, The Netherlands)
2. Department of Clinical Pharmacy, Ommelander Hospital Groningen (Winschoten, The Netherlands)
3. Department of Orthopaedic Surgery, Martini Hospital (Groningen, The Netherlands)
4. Department of Epidemiology, University of Groningen, University Medical Center Groningen, Groningen, The Netherlands
5. Department of Health Sciences, University of Groningen, University Medical Center Groningen (Groningen, The Netherlands)

Author responsible for correspondence: M. van Hulst PhD, PharmD

Martini Hospital
Department of Clinical Pharmacy and Toxicology
Van Swietenplein1
9728 NT Groningen, The Netherlands

UMCG
Department of Health Sciences
University of Groningen
University Medical Center Groningen
Groningen, the Netherlands

Tel.: ++31-50-5247382
e-mail: m.van.hulst@rug.nl

Trial registration number: ClinicalTrails.gov: NCT01431456

For peer review only

Abstract

Objectives: For the non-vitamin-K oral anticoagulants, data on bleeding when used for 42 days as thromboprophylaxis after TKA is scarce. This pilot study assessed feasibility of a multicentre randomised clinical trial to evaluate major and clinically relevant non-major bleeding during 42-day use of dabigatran, nadroparin and rivaroxaban after TKA.

Patients and Methods: In 70 weeks, 198 TKA patients were screened for eligibility in the Martini Hospital (Groningen, The Netherlands). Patients were randomly assigned to dabigatran (n=45), nadroparin (n=45), or rivaroxaban (n=48). The primary outcome was the combined end-point of major bleeding and clinically relevant non-major bleeding. Secondary endpoints of this study were the occurrence of clinical VTE (PE or DVT), compliance, duration of hospital stay, re-hospitalisation, adverse events and Knee injury and Osteoarthritis Outcome Score (KOOS).

Results: The primary outcome was observed in 33.3% (95%CI:20.0%–49.0%), 24.4% (95%CI:12.9%–39.5%) and 27.1% (95%CI:15.3%–41.8%) of patients who received dabigatran, nadroparin, or rivaroxaban, respectively (p=0.67). Major bleeding was found in two patients who received nadroparin (p=0.21). Clinically relevant non-major bleeding was observed in 33.3% (95%CI:20.0%–49.0%), 22.2% (95%CI:11.2%–37.1%), 27.1% (95%CI:15.3%-41.8%) for dabigatran, nadroparin and rivaroxaban, respectively (p=0.51). Wound hematoma was the most observed bleeding event. VTE was found in one patient who received dabigatran (p=0.65). The pre- and post-surgery KOOS questionnaires were available for 32 (71%), 35 (77%) and 35 (73%) patients for dabigatran, nadroparin and rivaroxaban, respectively. KOOS was highly variable, and no significant difference between treatment groups in mean improvement was observed.

Conclusions: A multicentre clinical trial may be feasible. However, investments will be substantial. No differences in major and non-major clinically relevant bleeding events were found between dabigatran, nadroparin and rivaroxaban during 42 days after TKA. KOOS may not be suitable to detect functional loss due to bleeding.

Article Summary

Strengths and limitations of this study

For peer review only

Strengths

- Direct comparison of dabigatran, rivaroxaban and nadroparin as thromboprophylaxis after total knee arthroplasty
- Evaluation of extended thromboprophylaxis for 42 days

Limitations

- Pilot-study, hence limited number of included patients
- Although aimed to enhance external validity, a large number of patients had to be excluded

For peer review only

Keywords: thrombosis, orthopaedic surgery, DOAC, NOAC, LMWH.

Word count

3,154

Introduction

After total knee arthroplasty (TKA) surgery, patients are at risk to develop venous thromboembolism (VTE). Without thromboprophylaxis, (venographic) DVT can be found in 40 – 80% of patients.[1,2] The risk of non-fatal and fatal PE for TKA surgery patients without thromboprophylaxis is 1.8% – 7.0% and 0.2% – 0.7%, respectively.[3] Hence, thromboprophylaxis is indicated for all patients undergoing TKA. Low molecular weight heparins (LMWHs) have become a gold standard in preventing VTE. Unfortunately, these anticoagulants have disadvantages such as, for example: a subcutaneous route of administration and risk of developing heparin-induced thrombocytopenia. These disadvantages can lead to low adherence of orthopaedic surgeons to the thromboprophylaxis guidelines and hamper compliance by patients.[4] During the last decades, oral anticoagulants were developed. Dabigatran, rivaroxaban, apixaban and edoxaban are four available non-vitamin-K oral anticoagulants (NOAC).

In clinical trials as well as in a real-world data study rivaroxaban was superior in preventing VTE compared to enoxaparin in TKA surgery patients.[5-7] However, compared to enoxaparin, rivaroxaban was associated with a slightly increased risk of postoperative bleeding and wound complications.[8] Clinical trials and subsequent meta-analyses showed that VTE risk and bleeding risk are similar for dabigatran compared to LMWH in TKA.[8-11]

The external validity of the rivaroxaban and dabigatran clinical trials may be limited for Dutch TKA patients. Next to that enoxaparin was used in the clinical trials, whereas in the Netherlands nadroparin is predominantly used, also the duration is extended in the Netherlands.[12] In the market authorisation for dabigatran and rivaroxaban the duration of VTE prevention is limited to 10 – 14 days. In our study, thromboprophylaxis was offered to patients for a period of 42 days after surgery in agreement with Dutch and regional guidelines.[12],[13] Moreover, clinical trials have demonstrated that a more prolonged prophylaxis with LMWH after hospital discharge significantly reduces the incidence of venographically detected DVT and PE after arthroplasty.[14,15]

For peer review only

The objective of our study was to compare the 42 day use of dabigatran, nadroparin and rivaroxaban on safety after TKA in a randomised open label pilot study by assessing the risk of major and clinically relevant non-major bleeding using a standardized model of bleeding definitions,[16] in order to obtain insight into the design of a multi-centre study investigating the safety of the new oral anticoagulants for prevention of venous thromboembolism after TKA in the Dutch setting. The rationale of our study was previously published.[17]

For peer review only

Methods

Study design

This study was an open label randomized pilot trial with a three-arm design, conducted at the Martini Hospital in Groningen, The Netherlands. This study was approved by the Medical Ethical committee "Regionale Toetsingscommissie Patiëntgebonden Onderzoek" (RTPO) in Leeuwarden, The Netherlands. This clinical trial was registered under NCT01431456 at clinicaltrials.gov. At the start of the study, an independent hospital pharmacist assigned the three treatment groups to a consecutive series of numbers, using a computer-generated randomised list. Before surgery, patients were included and randomly assigned to one of the three treatment groups (dabigatran, rivaroxaban or nadroparin). The allocation concealment was reached by using the sequentially numbered opaque sealed envelopes (SNOSE) method.[18] On enrolment, each patient was assigned to the next consecutive treatment number and the corresponding study medication was dispensed in an open-label fashion. Patients or the public were not involved in the design, or reporting, or dissemination plans of our research as this study is a pilot study. Patients were involved in the conduct of the trial by sharing experiences during the visits.

Study population

The target population consisted of TKA patients. Patients ≥ 18 years and weighing more than 40 kg who were scheduled for primary elective TKA and had provided signed informed consent were eligible for the study. Exclusion criteria included: a known inherited or acquired clinically significant active high risk of bleeding or bleeding disorder; major surgery, trauma, uncontrolled severe arterial hypertension, or myocardial infarction within the last three months; history of acute intracranial disease or haemorrhagic stroke; gastrointestinal or urogenital bleeding or ulcer disease within the last six months; cirrhotic patients with moderate hepatic impairment (aspartate or alanine aminotransferase levels higher than twice the upper limit of the normal range within the last month); severe renal insufficiency (creatinine clearance < 30 mL/min); other indication for treatment with anticoagulants; active malignant disease; pregnancy or breastfeeding. Patients received standard orthopaedic care

For peer review only

and physiotherapy according to the local standardised protocol. During the enrolment of the trial, fast-track knee replacement surgery was implemented.[19,20]

Treatment regimens

Patients were assigned to oral dabigatran etexilate 150 mg or 220 mg (two capsules, 75 mg or 110 mg) once daily, subcutaneous nadroparin 2850 IE = 0.3 ml once daily or 10 mg of oral rivaroxaban once daily. The first dose of dabigatran was one-half of subsequent doses (one capsule, 75 mg or 110 mg). Treatment was continued for a total of 42 days after TKA.

Outcome measures

The primary endpoint of this study was a major bleeding event and or clinically relevant non-major bleeding event within the 42-days treatment period. Any bleeding event that occurred during the 42-days treatment period, perceived by patient, researcher, nurse, orthopaedic surgeon or other health worker were registered and evaluated. A blinded independent expert adjudication committee classified all bleeding events according to international guidelines.[16] A major bleeding was defined as fatal bleeding, clinically overt bleeding associated with a decrease in the haemoglobin level of more than 20 g/l compared with the pre-randomisation level, clinically overt bleeding leading to transfusion of ≥2 units of whole blood or packed cells, critical bleeding (intracerebral, intraocular, intraspinal, pericardial or retroperitoneal), bleeding warranting treatment cessation, bleeding located at the surgical site and leading to re-operation or to any unusual medical intervention or procedure for relief (eg, draining or puncture of a haematoma at the surgical site, transfer to an ICU or emergency room). Clinically relevant non-major bleeding was defined as spontaneous skin haematoma >25cm^2, wound haematoma >100cm^2, spontaneous nose bleeding or gingival bleeding lasting longer than 5 min, spontaneous rectal bleeding creating more than a spot on toilet paper, macroscopic haematuria either spontaneous or, if associated with an intervention, lasting longer than 24 h, other bleeding events considered clinically relevant by the investigator not qualifying as a major bleeding.

For peer review only

Secondary endpoints of this study were the occurrence of clinical VTE (PE or DVT), compliance, duration of hospital stay, re-hospitalisation, adverse events and Knee injury and Osteoarthritis Outcome Score (KOOS).[21]

The duration of hospital stay and re-hospitalisation were retrieved from electronic medical records.

Compliance was measured by counting the number of doses left over after 42 days of treatment. In our study, patients were deemed compliant if patients didn't miss a single dose.

Adverse events were included in the analysis if the relationship with the study drug was possible, probable or highly probable.

The KOOS (www.koos.nu) is a 42-item self-administered questionnaire that includes five dimensions: pain, disease-related symptoms, activities of daily living function (ADL), sport and recreation function, and knee-related quality of life measured using a Likert scale (0 – 4 scale).[21] The KOOS was administered pre-surgery and six weeks after TKA. A patient with an improvement of ten points or more on a KOOS dimension after TKA was defined as responder.

Statistical analysis

The absolute bleeding risk was estimated as the percentage of patients with a bleed for each treatment group, with the associated 95% confidence intervals. Confidence intervals were derived using the (exact) Clopper-Pearson method. The risk of thromboembolic events was assessed using the same methodology. The analysis was based on the per-protocol population, i.e. all fully compliant patients that were evaluated 42 days after surgery. One-way analysis of variance (ANOVA) was used for analyses of continuous variables between multiple groups. If normality assumption was violated (Shapiro-Wilk test for normality), the non-parametric Kruskal Wallis test was used. Distribution of nominal variables for different groups was tested for statistical significance using the exact Chi-squared test. All statistical analyses were performed using IBM Statistics SPSS 25.0.0. Due to the explorative nature of this pilot study, all *p*-values will be interpreted as explorative rather than confirmative.[22] The expected sample size for the design of a two-arm clinical trial based on our findings was

For peer review only

calculated using the SampleSize4ClinicalTrials package in R (R Studio Version 1.3.959 / R version 4.0.3).

Results

Between July 2012 and November 2013, a total of 198 patients were assessed for eligibility of which 148 patients were randomised to treatment. In total 138 patients were included in the final analysis, see flow diagram (Figure 1). The three treatment groups were well balanced in terms of baseline demographics (Table 1). Of all patients, 63% were woman, with a mean age of 66 years and a mean weight of 89 kg. The mean time from end of surgery to the first study drug intake was 8 hours and 48 minutes. In total, 111 (80%) patients had fast-track TKA. Compared to patients in the other treatment groups, a larger share of patients in the dabigatran group had fast-track TKA. Thirty-three (24%) patients received a patella prosthesis during TKA. Compared to the nadroparin group, a larger share of patients in the rivaroxaban group and smaller share of patients in the dabigatran group received a new patella. Pre-surgery KOOS was well balanced between the three treatment groups.

Primary endpoints

The combined endpoint of major and clinically relevant non-major bleeding was observed in 15 of 45 (33.3%; 95% CI 20.0% – 49.0%) patients who received dabigatran, 11 of 45 (24.4%; 95% CI 12.9% – 39.5%) patients who received nadroparin and 13 of 48 (27.1%; 95% CI 15.3% – 41.8%) patients who received rivaroxaban. There was no significant difference in these bleeding events between dabigatran, rivaroxaban and nadroparin (p = 0.67), see Table 2.

Major bleeding events occurred in none of 45 (0%; 95% CI 0% – 7.9%) patients in the dabigatran group, two of 45 (4.4%; 95% CI 0.5% – 15.1%) patients in the nadroparin group and none of 48 (0%; 95% CI 0% – 7.4%) patients in the rivaroxaban group. One of these events was a clinically overt bleeding from the wound leading to transfusion of 2 or more units of whole blood or packed cells and one was adjudicated as a subdural hematoma caused by a fall. There was no difference in major bleeding events between dabigatran, nadroparin and rivaroxaban.

For peer review only

Clinically relevant non-major bleeding events were observed in 15 patients who received dabigatran (33.3%; 95% CI 20.0% – 49.0%), 10 patients who received nadroparine (22.2%; 95% CI 11.2% – 37.1%),) and 13 patients who received rivaroxaban (27.1%; 95% CI 15.3% – 41.8%). Most of these clinically relevant non-major bleeding events were wound hematomas. There was no difference in clinically relevant non-major bleeding events between dabigatran, rivaroxaban and nadroparin. The last major or clinically relevant non-major bleeding event was observed a median seven, seven and six days post-operatively, for dabigatran, nadroparin and rivaroxaban, respectively. In the period between 14 and 42 days after surgery, one major or clinically relevant non-major bleeding event was observed in all treatment groups. The overall risk of major and clinically relevant non-major bleeding was 39 events in a total intention to treat population of 148 randomized patients, i.e. a proportion of 0.26. Using a relative risk difference of 10%, i.e. an absolute difference of 2.6%, we estimated that 4,468 patients per treatment group would be needed in a two-arm non-inferiority design with a statistical power of 80% and a two-sided type I error rate of 2.5%.

Secondary endpoints

The secondary endpoints of this study were: occurrence of clinical VTE, hospital stay, re-admission, adverse events, compliance and KOOS, see Table 2 and 3. Clinical VTE occurred in one of 45 patients receiving dabigatran and in none receiving rivaroxaban or nadroparin. Unfortunately, this patient with VTE developed PE and died. The median duration of hospital stay was three, three and four days for dabigatran, nadroparine and rivaroxaban, respectively. Re-admission within three months post-surgery was not different between treatment groups. The number of patients re-admitted within three months was two (4.4%), four, (8.9%) and three (6.3%) patients, for dabigatran, nadroparin and rivaroxaban, respectively. Adverse events were reported in nine (20%), two (4.4%) and five (10.4%) patients for dabigatran, nadroparin and rivaroxaban, respectively. Five patients in the dabigatran group (11.1%) experienced gastro-intestinal adverse effects such as abdominal pain, diarrhoea and/or indigestion. In the rivaroxaban group, gastro-intestinal adverse effects were found in two patients (4.4%).

For peer review only

In the dabigatran group, 27 of 44 patients (61.4%) were compliant versus 25 of 44 patients (56.8%) in the nadroparine group and 25 of 48 patients in the rivaroxaban group (52.1%). Compliance was not different between treatment groups. Also, the number of missed days of treatment was not different between treatment groups. In the period between the 42-day follow-up and three months post-surgery, we observed no additional bleeding and VTE events.

The pre- and post-surgery KOOS questionnaires were available for 32 (71%), 35 (77%) and 35 (73%) patients for dabigatran, nadroparin and rivaroxaban, respectively (Table 3). No difference between treatment groups in mean improvement was observed for the KOOS dimensions pain, symptoms, ADL, sport and recreation and quality of life. Also, the proportion of responders, patients with a ten point or more improvement on a KOOS dimension, was not different between dabigatran, nadroparin and rivaroxaban. The standard deviation in KOOS was mostly equal or even higher than the mean.

For peer re

Discussion

After knee replacement, when bleeding in the replaced joint can have serious consequences, the balance between inducing bleeding and preventing VTE is pivotal in thromboprophylaxis. In this study, we compared bleeding complications of dabigatran, rivaroxaban and nadroparin when used to prevent VTE after TKA surgery. This exploratory study shows that there was no significant difference in major and clinically relevant bleeding between dabigatran, rivaroxaban and nadroparin. The bleeding risk we found is higher than observed in previous knee replacement studies. In such studies a risk of non-major bleeding events of between 1% and 6.8% was observed.[5,6,23–25] In our study, the risk of bleeding events is higher mainly due to the risk of wound haematomas > 100 cm^2. A possible explanation for the difference in occurrence of clinically relevant non-major bleeding events is our strict adherence to EMA bleeding definitions.[16 24] For instance, in the RECORD 3 and RECORD 4 rivaroxaban trials, surgical site and wound haematomas were not reported.[5,6,8,24] In the EPCAT II trial, extended venous thromboembolism prophylaxis comparing rivaroxaban to aspirin following total hip and knee arthroplasty, only infected wound haematomas were considered clinically relevant.[25]

The market authorisation for the prevention of venous thromboembolism after total knee arthroplasty for dabigatran and rivaroxaban is 10-14 days. In our study, patients received thromboprophylaxis for 42 days. Most bleeding events occurred during the first 14 days post-surgery. Notably, bleeding risks after the first 14 days use of dabigatran, nadroparin and rivaroxaban were low and not different between treatment groups. Based on our pilot-study, a meta-analysis of dabigatran and rivaroxaban in TKA and the EPCAT II trial, it is expected that large numbers of patients have to be included in a clinical trial to detect a clinically relevant difference in major and clinically relevant non-major bleeding between NOACs and LMWHs when used for 42 days.[8,25] Therefore, costs of conducting a multicentre clinical trial based on the evaluated protocol and a formal power calculation are expected to be very high.

As expected, the number of symptomatic VTE cases was very low. Unfortunately, one case of PE occurred in the dabigatran group, leading to the death of the patient.

It is generally assumed that patients would prefer the oral route to subcutaneous injections. However, it is unknown if the route of administration has an impact on patient compliance. In our pilot-study, we found no clear indication for a difference in compliance between rivaroxaban, nadroparin and dabigatran.

Although we found no differences in the number of patients with adverse reactions, the number of patients with gastro-intestinal adverse reaction in the dabigatran group appeared to be higher than in the other treatment groups. Dabigatran is also in other clinical trials associated with gastro-intestinal adverse effects.[26] Between dabigatran, nadroparin and rivaroxaban no difference in patient reported outcomes as assessed with the KOOS was found. The hypothesis was that joint haemorrhages could impair subjective improvement after TKA as assessed by KOOS. Our pilot study shows that it is feasible to assess KOOS in a study evaluating bleeding complications of thromboprophylaxis. However, variability in KOOS is probably too high to detect differences in KOOS in a future multicentre clinical trial.

For peer review only

The results of this pilot study should be interpreted with caution due to some limitations. Firstly, as a pilot-study, the size of the study population is limited. Although our aim was to conduct a pilot study to design a multicentre study with an improved external validity compared to available randomised clinical trials, we had to exclude a large number of patients some of who will receive thromboprophylaxis with NOACs in regular clinical practice. Additionally, we had to exclude patients with a high risk of bleeding who may receive thromboprophylaxis with NOACs in regular clinical practice.

Conclusion

A multicentre clinical trial may be feasible based on the recruitment speed and satisfaction with the trial protocol. No differences in major and non-major clinically relevant bleeding events were found in a pilot-study of the head-to-head comparison of dabigatran, nadroparin and rivaroxaban as 42-day thromboprophylaxis in TKA. After the first 14 days use of dabigatran, nadroparin or rivaroxaban, bleeding risks were low and not different. This finding implies that conducting a multicentre trial will require substantial investment to achieve an adequate number of evaluated patients. Furthermore, due to the high variability in the KOOS, this outcome score may not be suitable to detect functional loss due to bleeding in a future clinical trial.

Acknowledgements

The adjudication committee (mrs A. van der Aart, hospital pharmacist; mrs A. van der Velden, internist-haematologist / oncologist and dr. R.W. Brouwer, orthopaedic surgeon) is thanked for the careful assessment of bleeding events. Dr Brouwer joint the writing committee after adjudication of bleeding events. Mrs Roya Sorghabi is thanked for clinical trial management and data acquisition. Nurses and doctors of the orthopaedic surgery department are thanked for screening patients for bleeding events.

Author Contributions

LV, MH, CG and JR conceived the idea of the study and were responsible for the design of the study. NV, LV and MH performed the statistical analysis for the study. The initial draft of

For peer review only

the manuscript was prepared by LV, MS, MH, JR, RB and NV and then circulated repeatedly among all authors for critical revision. LV and MS were responsible for the acquisition of the data and MH and NV contributed to the interpretation of the results. LV was the coordinator of the study and lead writer. All authors helped plan the study, evolve analysis plans and critically revise successive drafts of the manuscript.

Funding

This research received no specific grant from any funding agency in the public, commercial or not-for-profit sectors.

Competing interests

MH reports grants from Bayer and personal fees from Boehringer Ingelheim during the conduct of the study. Other authors have nothing to report.

Data sharing

Data can made be available upon request to the corresponding author.

For peer review only

Table 1. Patient characteristics

	Dabigatran n = 45	Nadroparine n = 45	Rivaroxaban n = 48	Total n = 138
Female n (%)	26 (58%)	31 (69%)	30 (63%)	87 (63%)
Age (year) [±SD]	66±7.9	67±10	65±7.5	66±8.7
Weight (kg) [±SD]	88±12	89±18	90±21	89±17
Fast-track surgery (%)	41 (91%)	35 (78%)	35 (73%)	111 (80%)
Received new patella (%)	7 (16%)	10 (22%)	16 (33%)	33 (24%)
History of VTE (%)	1 (2.2%)	1 (2.2%)	2 (4.2%)	4 (2.9%)
Time to first dose (h) [±SD]	8.6±2.3	9.0±2.2	8.8±2.1	8.8±2.2
Pre-surgery KOOS [±SD]				
- pain {n}	47±15 {32}	48±20 {35}	51±21 {35}	49±19 {102}
- symptoms {n}	55±16 {32}	49±18 {35}	50±18 {35}	51±17 {102}
- ADL {n}	52±15 {32}	48±19 {35}	56±19 {35}	52±18 {102}
- sport and recreation {n}	23±25 {29}	24±28 {31}	22±22 {29}	23±25 {89}
- quality of life {n}	30±12 {31}	30±16 {35}	30±15 {35}	30±14 {101}

ADL: activities of daily living function; KOOS: Knee injury and Osteoarthritis Outcome Score; SD: standard deviation; VTE: venous thromboembolism.

For peer review only

Table 2. Primary and secondary outcomes of thromboprophylaxis with dabigatran, nadroparin or rivaroxaban after total knee arthroplasty

	Dabigatran n = 45		Nadroparin n = 45		Rivaroxaban n = 48		*p*-value
	n	%	*n*	%	*n*	%	
Primary outcome							
Major and clinically relevant non-major bleeding	15	33.3	11	24.4	13	27.1	$p = 0.67$
Major bleedings	0	0	2	4.4	0	0	$p = 0.21$
Fatal bleeding	0	0	0	0	0	0	
Clinically overt bleeding, Hb decrease>20 g/l	0	0	0	0	0	0	
Clinically overt bleeding, transfusion	0	0	1	2.2	0	0	$p = 0.65$
Critical bleeding	0	0	1*	2.2	0	0	$p = 0.65$
Bleeding warranting treatment cessation	0	0	1*	2.2	0	0	$p = 0.65$
Bleeding located at surgical site	0	0	0	0	0	0	
Clinically relevant non-major bleeding	15	33.3	10	22.2	13	27.1	$p = 0.51$
Spontaneous skin haematoma >25 cm2	1	2.2	0	0	0	0	$p = 0.65$
Wound haematoma >100 cm2	14	31.1	9	20.0	12	25.0	$p = 0.49$
Spontaneous nose bleeding > 5 min	0	0	1	2.2	0	0	$p = 0.65$
Spontaneous rectal bleeding > 1 spot	0	0	0	0	0	0	
Macroscopic haematuria > 24 h	0	0	0	0	1	2.1	$p = 1.0$
Other bleeding event (not major)	0	0	0	0	0	0	
Last primary outcome (median days after surgery) [range]	7 [1 – 21]		7 [2 – 26]		6 [2 – 16]		$p = 0.32$
Patients with primary outcome more than 14 days after surgery (%)	1	2.2	1	2.2%	1	2.1	$p = 1.0$
Secondary outcome							
VTE	1*	2.2	0	0	0	0	$p = 0.65$
Death due to VTE	1*		0		0		
DVT	0		0		0		
PE	1*		0		0		
Hospital stay (median, days) [range]	3 [2 – 9]		3 [2 – 10]		4 [2 – 28]		$p = 0.098$
Re-admission within three months (%)	2 (4.4%)		4 (8.9%)		3 (6.3%)		$p = 0.77$
Patients with adverse reactions (%)	9 (20.0%)		2 (4.4%)		5 (10.4%)		$p = 0.072$
Number of patient compliant (%)	27 of 44 (61.4%)†		25 of 44 (56.8%)†		25 (52.1%)		$p = 0.66$
Days of missed dosages (median, days) [range]	0 [0 — 7]; n=42		0 [0 – 23]; n=40		0 [0 – 4]; n=40		$p = 0.73$

VTE: venous thromboembolism; PE: pulmonary embolism; DVT: deep venous thrombosis; SD: standard deviation;
*identical patient; † one missing value. Due to the explorative nature of this pilot study, all p-values are explorative rather than confirmative and are not corrected for multiple testing.

For peer review only

Table 3. Knee injury and Osteoarthritis Outcome Score (KOOS) for patients with dabigatran, nadroparin or rivaroxaban thromboprophylaxis after total knee arthroplasty. Difference between six weeks post-surgery and pre-surgery KOOS and proportion of patients with 10 points or more improvement in KOOS between six weeks post-surgery and pre-surgery.

	Dabigatran	Nadroparin	Rivaroxaban	*p*-value
Pain	n = 32	n = 35	n = 35	
Difference six weeks post-surgery and pre-surgery (mean±SD)	25±26	19±22	22±23	p=0.51
Patients with ≥10 points improvement post- vs pre-surgery (n; %)	22; 69%	26; 74%	27; 77%	p=0.78
Symptoms	n = 32	n = 35	n = 35	
Difference six weeks post-surgery and pre-surgery (mean±SD)	12±20	14±20	16±19	p=0.63
Patients with ≥10 points improvement post- vs pre-surgery (n, %)	17; 53%	20; 57%	22; 63%	p=0.72
ADL	n = 32	n = 35	n = 35	
Difference six weeks post-surgery and pre-surgery (mean±SD)	25±20	23±21	21±17	p=0.75
Patients with ≥10 points improvement post- vs pre-surgery (n, %)	23; 72%	25; 71%	27; 77%	p=0.88
Sport and recreation	n = 29	n = 31	n = 29	
Difference six weeks post-surgery and pre-surgery (mean±SD)	24±38	25±41	28±33	p=0.89
Patients with ≥10 points improvement post- vs pre-surgery (n, %)	17; 59%	21; 68%	23;79%	p=0.24
Quality of Life	n = 31	n = 35	n = 35	
Difference six weeks post-surgery and pre-surgery (mean±SD)	26±27	18±20	22±21	p=0.39
Patients with ≥10 points improvement post- vs pre-surgery (n, %)	24;77%	23;66%	23; 66%	p=0.55

ADL: activities of daily living function; SD: standard deviation. Due to the explorative nature of this pilot study, all p-values are explorative rather than confirmative and are not corrected for multiple testing.

For peer review only - http://bmjopen.bmj.com/site/about/guidelines.xhtml

Figure 1. Flow diagram. AF: atrial fibrillation; ALT: alanine aminotransferase; AST: aspartate aminotransferase.

For peer review only

Reference list

1. Warwick D, Friedman RJ, Agnelli G, et al. Insufficient duration of venous thromboembolism prophylaxis after total hip or knee replacement when compared with the time course of thromboembolic events: findings from the Global Orthopaedic Registry. *The Journal of bone and joint surgery British volume* 2007; **89**(6): 799-807.
2. White RH. The epidemiology of venous thromboembolism. *Circulation* 2003; **107**(23 Suppl 1): I4-8.
3. Falck-Ytter Y, Francis CW, Johanson NA, et al. Prevention of VTE in orthopedic surgery patients: Antithrombotic Therapy and Prevention of Thrombosis, 9th ed: American College of Chest Physicians Evidence-Based Clinical Practice Guidelines. *Chest* 2012; **141**(2 Suppl): e278S-325S.
4. Rachidi S, Aldin ES, Greenberg C, Sachs B, Streiff M, Zeidan AM. The use of novel oral anticoagulants for thromboprophylaxis after elective major orthopedic surgery. *Expert review of hematology* 2013; **6**(6): 677-95.
5. Lassen MR, Ageno W, Borris LC, et al. Rivaroxaban versus enoxaparin for thromboprophylaxis after total knee arthroplasty. *The New England journal of medicine* 2008; **358**(26): 2776-86.
6. Turpie AG, Lassen MR, Davidson BL, et al. Rivaroxaban versus enoxaparin for thromboprophylaxis after total knee arthroplasty (RECORD4): a randomised trial. *Lancet* 2009; **373**(9676): 1673-80.
7. Turpie AG, Haas S, Kreutz R, et al. A non-interventional comparison of rivaroxaban with standard of care for thromboprophylaxis after major orthopaedic surgery in 17,701 patients with propensity score adjustment. *Thrombosis and haemostasis* 2014; **111**(1): 94-102.
8. Gomez-Outes A, Terleira-Fernandez AI, Suarez-Gea ML, Vargas-Castrillon E. Dabigatran, rivaroxaban, or apixaban versus enoxaparin for thromboprophylaxis after total hip or knee replacement: systematic review, meta-analysis, and indirect treatment comparisons. *BMJ* 2012; **344**: e3675.
9. Huisman MV, Quinlan DJ, Dahl OE, Schulman S. Enoxaparin versus dabigatran or rivaroxaban for thromboprophylaxis after hip or knee arthroplasty: Results of separate pooled analyses of phase III multicenter randomized trials. *Circulation Cardiovascular quality and outcomes* 2010; **3**(6): 652-60.
10. Friedman RJ, Dahl OE, Rosencher N, et al. Dabigatran versus enoxaparin for prevention of venous thromboembolism after hip or knee arthroplasty: a pooled analysis of three trials. *Thrombosis research* 2010; **126**(3): 175-82.
11. Venker BT, Ganti BR, Lin H, Lee ED, Nunley RM, Gage BF. Safety and Efficacy of New Anticoagulants for the Prevention of Venous Thromboembolism After Hip and Knee Arthroplasty: A Meta-Analysis. *The Journal of arthroplasty* 2017; **32**(2): 645-52.
12. Vereniging NI. Keuze en duur van tromboseprofylaxe bij grote orthopedische en traumatologische ingrepen. https://richtlijnendatabase.nl/richtlijn/antitrombotisch_beleid/preventie_vte/keuze_en_duur_profylaxe_grote_ingrepen.html#verantwoording (accessed 13-02-2018).
13. Ettema HB, Mulder MC, Nurmohamed MT, Buller HR, Verheyen CC. Dutch orthopedic thromboprophylaxis: a 5-year follow-up survey. *Acta orthopaedica* 2009; **80**(1): 109-12.
14. White RH, Romano PS, Zhou H, Rodrigo J, Bargar W. Incidence and time course of thromboembolic outcomes following total hip or knee arthroplasty. *Archives of internal medicine* 1998; **158**(14): 1525-31.

For peer review only

15. Bergqvist D, Benoni G, Bjorgell O, et al. Low-molecular-weight heparin (enoxaparin) as prophylaxis against venous thromboembolism after total hip replacement. *The New England journal of medicine* 1996; **335**(10): 696-700.
16. (EMA) EMA. Guideline on clinical investigation of medicinal products for the prophylaxis of high intra- and postoperative venous thromboembolic risk. *CPMP/EWP/707/98* 2007; **15-11-2007**.
17. Veen L, van Raay JJ, Gerritsma-Bleeker CL, Veeger NJ, Hulst M. Direct treatment comparison of DAbigatran and RIvaroxaban versus NAdroparin in the prevention of venous thromboembolism after total knee arthroplasty surgery: design of a randomised pilot study (DARINA). *BMJ open* 2013; **3**(1).
18. Doig GS, Simpson F. Randomization and allocation concealment: a practical guide for researchers. *Journal of critical care* 2005; **20**(2): 187-91; discussion 91-3.
19. Husted H, Jensen CM, Solgaard S, Kehlet H. Reduced length of stay following hip and knee arthroplasty in Denmark 2000-2009: from research to implementation. *Archives of orthopaedic and trauma surgery* 2012; **132**(1): 101-4.
20. Van Egmond JC, Verburg H, Mathijssen NM. The first 6 weeks of recovery after total knee arthroplasty with fast track. *Acta orthopaedica* 2015; **86**(6): 708-13.
21. de Groot IB, Favejee MM, Reijman M, Verhaar JA, Terwee CB. The Dutch version of the Knee Injury and Osteoarthritis Outcome Score: a validation study. *Health and quality of life outcomes* 2008; **6**: 16.
22. Julious SA. Sample size of 12 per group rule of thumb for a pilot study. *Pharmaceutical Statistics* 2005; **4**(4): 5.
23. Eriksson BI, Dahl OE, Rosencher N, et al. Oral dabigatran etexilate vs. subcutaneous enoxaparin for the prevention of venous thromboembolism after total knee replacement: the RE-MODEL randomized trial. *Journal of thrombosis and haemostasis : JTH* 2007; **5**(11): 2178-85.
24. Dahl OE, Quinlan DJ, Bergqvist D, Eikelboom JW. A critical appraisal of bleeding events reported in venous thromboembolism prevention trials of patients undergoing hip and knee arthroplasty. *Journal of thrombosis and haemostasis : JTH* 2010; **8**(9): 1966-75.
25. Anderson DR, Dunbar M, Murnaghan J, et al. Aspirin or Rivaroxaban for VTE Prophylaxis after Hip or Knee Arthroplasty. *The New England journal of medicine* 2018; **378**(8): 699-707.
26. Connolly SJ, Ezekowitz MD, Yusuf S, et al. Dabigatran versus warfarin in patients with atrial fibrillation. *The New England journal of medicine* 2009; **361**(12): 1139-51.

For peer review only

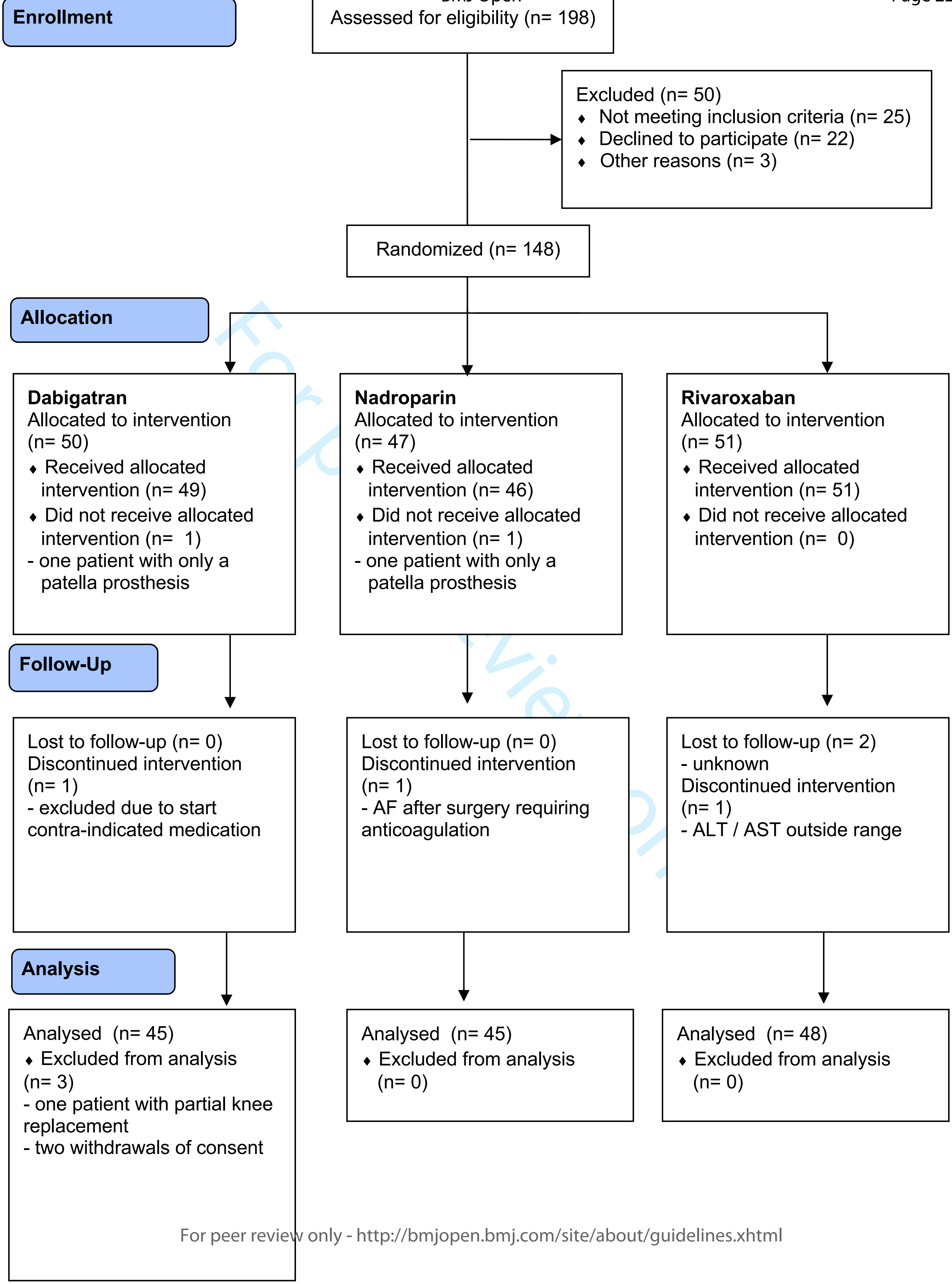

For peer review only

Reporting checklist for randomised trial.

Based on the CONSORT guidelines.

Instructions to authors

Complete this checklist by entering the page numbers from your manuscript where readers will find each of the items listed below.

Your article may not currently address all the items on the checklist. Please modify your text to include the missing information. If you are certain that an item does not apply, please write "n/a" and provide a short explanation.

Upload your completed checklist as an extra file when you submit to a journal.

In your methods section, say that you used the CONSORTreporting guidelines, and cite them as:

Schulz KF, Altman DG, Moher D, for the CONSORT Group. CONSORT 2010 Statement: updated guidelines for reporting parallel group randomised trials

		Reporting Item	Page Number
**Title and Abstract**			
Title	#1a	Identification as a randomized trial in the title.	Page 1
Abstract	#1b	Structured summary of trial design, methods, results, and conclusions	Page 2
**Introduction**			
Background and objectives	#2a	Scientific background and explanation of rationale	Page 4
Background and objectives	#2b	Specific objectives or hypothesis	Page 4
**Methods**			
Trial design	#3a	Description of trial design (such as parallel, factorial) including allocation ratio.	Page 6
Trial design	#3b	Important changes to methods after trial commencement (such as eligibility criteria), with reasons	Page 6

For peer review only

Participants	#4a	Eligibility criteria for participants	Page 6
Participants	#4b	Settings and locations where the data were collected	Page 6
Interventions	#5	The experimental and control interventions for each group with sufficient details to allow replication, including how and when they were actually administered	Page 6 – 7
Outcomes	#6a	Completely defined prespecified primary and secondary outcome measures, including how and when they were assessed	Page 7
Sample size	#7a	How sample size was determined.	Page 8
Sample size	#7b	When applicable, explanation of any interim analyses and stopping guidelines	Page 8
Randomization - Sequence generation	#8a	Method used to generate the random allocation sequence.	
Page 6			
Randomization - Sequence generation	#8b	Type of randomization; details of any restriction (such as blocking and block size)	
Page 6			
Randomization - Allocation concealment mechanism	#9	Mechanism used to implement the random allocation sequence (such as sequentially numbered containers), describing any steps taken to conceal the sequence until interventions were assigned	Page 6
Randomization - Implementation	#10	Who generated the allocation sequence, who enrolled participants, and who assigned participants to interventions	Page 6
Blinding	#11a	If done, who was blinded after assignment to interventions (for example, participants, care providers, those assessing outcomes) and how.	Page 6
Blinding	#11b	If relevant, description of the similarity of interventions	Page 6
Statistical methods	#12a	Statistical methods used to compare groups for primary and secondary outcomes	Page 8
Statistical methods	#12b	Methods for additional analyses, such as subgroup analyses and adjusted analyses	Page 8

For peer review only

Outcomes	#6b	Any changes to trial outcomes after the trial commenced, with reasons	Page 7
**Results**			
Participant flow diagram (strongly recommended)	#13a	For each group, the numbers of participants who were randomly assigned, received intended treatment, and were analysed for the primary outcome	Figure 1
Participant flow	#13b	For each group, losses and exclusions after randomization, together with reason	Figure 1
Recruitment	#14a	Dates defining the periods of recruitment and follow-up	Page 8
Recruitment	#14b	Why the trial ended or was stopped	n/a
Baseline data	#15	A table showing baseline demographic and clinical characteristics for each group	Page 15, table 1.
Numbers analysed	#16	For each group, number of participants (denominator) included in each analysis and whether the analysis was by original assigned groups	Figure 1
Outcomes and estimation	#17a	For each primary and secondary outcome, results for each group, and the estimated effect size and its precision (such as 95% confidence interval)	Page 9
Outcomes and estimation	#17b	For binary outcomes, presentation of both absolute and relative effect sizes is recommended	Page 9
Ancillary analyses	#18	Results of any other analyses performed, including subgroup analyses and adjusted analyses, distinguishing pre-specified from exploratory	Page 9 – 11
Harms	#19	All important harms or unintended effects in each group (For specific guidance see CONSORT for harms)	Page 10
**Discussion**			
Limitations	#20	Trial limitations, addressing sources of potential bias, imprecision, and, if relevant, multiplicity of analyses	Page 11
Interpretation	#22	Interpretation consistent with results, balancing benefits and harms, and considering other relevant evidence	Page 11
Registration	#23	Registration number and name of trial registry	Page 1

For peer review only

Other Information

Protocol	#24	Where the full trial protocol can be accessed, if available	Page 5 (ref 17) and Appendix
Funding	#25	Sources of funding and other support (such as supply of drugs), role of funders	Page 13

Notes:

- 15: Page 15, table 1.
- 18: Page 9 – 11
- 24: Page 5 (ref 17) and Appendix The CONSORT checklist is distributed under the terms of the Creative Commons Attribution License CC-BY. This checklist was completed on 24. April 2020 using https://www.goodreports.org/, a tool made by the EQUATOR Network in collaboration with Penelope.ai

For peer review only

Bleeding complications of thromboprophylaxis with dabigatran, nadroparin or rivaroxaban for six weeks after total knee arthroplasty surgery – a randomised pilot study

Journal:	*BMJ Open*
Manuscript ID	bmjopen-2020-040336.R2
Article Type:	Original research
Date Submitted by the Author:	16-Dec-2020
Complete List of Authors:	van der Veen, Lucia; Martini Hospital, Clinical Pharmacy; Ommelander Hospital Groningen, Department of Clinical Pharmacy<br>Segers, Marijn; Martini Hospital, Department of Clinical Pharmacy and Toxicology<br>van Raay, Jos; Martini Hospital, Department of Orthopaedic Surgery<br>Gerritsma-Bleeker, Carina; Martini Hospital, Department of Orthopaedic Surgery<br>Brouwer, Reinoud; Martini Hospital, Orthopaedic surgery<br>Veeger, Nic; Universitair Medisch Centrum Groningen, Department of Epidemiology<br>van Hulst, Marinus; Martini Hospital, Clinical Pharmacy; UMCG, Department of Health Sciences
**Primary Subject Heading**:	Pharmacology and therapeutics
Secondary Subject Heading:	Surgery, Haematology (incl blood transfusion)
Keywords:	Thromboembolism < CARDIOLOGY, Anticoagulation < HAEMATOLOGY, Knee < ORTHOPAEDIC & TRAUMA SURGERY, Bleeding disorders & coagulopathies < HAEMATOLOGY

I, the Submitting Author has the right to grant and does grant on behalf of all authors of the Work (as defined in the below author licence), an exclusive licence and/or a non-exclusive licence for contributions from authors who are: i) UK Crown employees; ii) where BMJ has agreed a CC-BY licence shall apply, and/or iii) in accordance with the terms applicable for US Federal Government officers or employees acting as part of their official duties; on a worldwide, perpetual, irrevocable, royalty-free basis to BMJ Publishing Group Ltd ("BMJ") its licensees and where the relevant Journal is co-owned by BMJ to the co-owners of the Journal, to publish the Work in this journal and any other BMJ products and to exploit all rights, as set out in our licence.

The Submitting Author accepts and understands that any supply made under these terms is made by BMJ to the Submitting Author unless you are acting as an employee on behalf of your employer or a postgraduate student of an affiliated institution which is paying any applicable article publishing charge ("APC") for Open Access articles. Where the Submitting Author wishes to make the Work available on an Open Access basis (and intends to pay the relevant APC), the terms of reuse of such Open Access shall be governed by a Creative Commons licence – details of these licences and which Creative Commons licence will apply to this Work are set out in our licence referred to above.

Other than as permitted in any relevant BMJ Author's Self Archiving Policies, I confirm this Work has not been accepted for publication elsewhere, is not being considered for publication elsewhere and does not duplicate material already published. I confirm all authors consent to publication of this Work and authorise the granting of this licence.

For peer review only

Bleeding complications of thromboprophylaxis with dabigatran, nadroparin or rivaroxaban for six weeks after total knee arthroplasty surgery – a randomised pilot study

Lucia van der Veen[1,2], Marijn Segers[1] Jos JAM van Raay[3], Carina L E Gerritsma-Bleeker[3], Reinoud W. Brouwer[3], Nic JGM Veeger[4], Marinus van Hulst[1,5]

1. Department of Clinical Pharmacy and Toxicology, Martini Hospital (Groningen, The Netherlands)
2. Department of Clinical Pharmacy, Ommelander Hospital Groningen (Winschoten, The Netherlands)
3. Department of Orthopaedic Surgery, Martini Hospital (Groningen, The Netherlands)
4. Department of Epidemiology, University of Groningen, University Medical Center Groningen, Groningen, The Netherlands
5. Department of Health Sciences, University of Groningen, University Medical Center Groningen (Groningen, The Netherlands)

Author responsible for correspondence: M. van Hulst PhD, PharmD

Martini Hospital
Department of Clinical Pharmacy and Toxicology
Van Swietenplein1
9728 NT Groningen, The Netherlands

UMCG
Department of Health Sciences
University of Groningen
University Medical Center Groningen
Groningen, the Netherlands

Tel.: ++31-50-5247382
e-mail: m.van.hulst@rug.nl

Trial registration number: ClinicalTrails.gov: NCT01431456

For peer review only

Abstract

Objectives: For the non-vitamin-K oral anticoagulants, data on bleeding when used for 42 days as thromboprophylaxis after TKA is scarce. This pilot study assessed feasibility of a multicentre randomised clinical trial to evaluate major and clinically relevant non-major bleeding during 42-day use of dabigatran, nadroparin and rivaroxaban after TKA.

Patients and Methods: In 70 weeks, between July 2012 and November 2013, 198 TKA patients were screened for eligibility in the Martini Hospital (Groningen, The Netherlands). Patients were randomly assigned to dabigatran (n=45), nadroparin (n=45), or rivaroxaban (n=48). The primary outcome was the combined end-point of major bleeding and clinically relevant non-major bleeding. Secondary endpoints of this study were the occurrence of clinical VTE (PE or DVT), compliance, duration of hospital stay, re-hospitalisation, adverse events and Knee injury and Osteoarthritis Outcome Score (KOOS).

Results: The primary outcome was observed in 33.3% (95%CI:20.0%–49.0%), 24.4% (95%CI:12.9%–39.5%) and 27.1% (95%CI:15.3%–41.8%) of patients who received dabigatran, nadroparin, or rivaroxaban, respectively (p=0.67). Major bleeding was found in two patients who received nadroparin (p=0.21). Clinically relevant non-major bleeding was observed in 33.3% (95%CI:20.0%–49.0%), 22.2% (95%CI:11.2%–37.1%), 27.1% (95%CI:15.3%-41.8%) for dabigatran, nadroparin and rivaroxaban, respectively (p=0.51). Wound hematoma was the most observed bleeding event. VTE was found in one patient who received dabigatran (p=0.65). The pre- and post-surgery KOOS questionnaires were available for 32 (71%), 35 (77%) and 35 (73%) patients for dabigatran, nadroparin and rivaroxaban, respectively. KOOS was highly variable, and no significant difference between treatment groups in mean improvement was observed.

Conclusions: A multicentre clinical trial may be feasible. However, investments will be substantial. No differences in major and non-major clinically relevant bleeding events were found between dabigatran, nadroparin and rivaroxaban during 42 days after TKA. KOOS may not be suitable to detect functional loss due to bleeding.

Article Summary

Strengths and limitations of this study

For peer review only

Strengths

- In this study, dabigatran, rivaroxaban and nadroparin were directly compared as thromboprophylaxis after total knee arthroplasty surgery
- We evaluated extended thromboprophylaxis for 42 days after total knee arthroplasty surgery

Limitations

- Pilot-study, hence limited number of patients were evaluated

Keywords: thrombosis, orthopaedic surgery, DOAC, NOAC, LMWH.

Word count

3,195

For peer review only

Introduction

After total knee arthroplasty (TKA) surgery, patients are at risk to develop venous thromboembolism (VTE). Without thromboprophylaxis, (venographic) DVT can be found in 40 – 80% of patients.[1,2] The risk of non-fatal and fatal PE for TKA surgery patients without thromboprophylaxis is 1.8% – 7.0% and 0.2% – 0.7%, respectively.[3] Hence, thromboprophylaxis is indicated for all patients undergoing TKA. Low molecular weight heparins (LMWHs) have become a gold standard in preventing VTE. Unfortunately, these anticoagulants have disadvantages such as, for example: a subcutaneous route of administration and risk of developing heparin-induced thrombocytopenia. These disadvantages can lead to low adherence of orthopaedic surgeons to the thromboprophylaxis guidelines and hamper compliance by patients.[4] During the last decades, oral anticoagulants were developed. Dabigatran, rivaroxaban, apixaban and edoxaban are four available non-vitamin-K oral anticoagulants (NOAC).

In clinical trials as well as in a real-world data study rivaroxaban was superior in preventing VTE compared to enoxaparin in TKA surgery patients.[5-7] However, compared to enoxaparin, rivaroxaban was associated with a slightly increased risk of postoperative bleeding and wound complications.[8] Clinical trials and subsequent meta-analyses showed that VTE risk and bleeding risk are similar for dabigatran compared to LMWH in TKA.[8-11]

The external validity of the rivaroxaban and dabigatran clinical trials may be limited for Dutch TKA patients. Next to that enoxaparin was used in the clinical trials, whereas in the Netherlands nadroparin is predominantly used, also the duration is extended in the Netherlands.[12] In the market authorisation for dabigatran and rivaroxaban the duration of VTE prevention is limited to 10 – 14 days. In our study, thromboprophylaxis was offered to patients for a period of 42 days after surgery in agreement with Dutch and regional guidelines.[12],[13] Moreover, clinical trials have demonstrated that a more prolonged prophylaxis with LMWH after hospital discharge significantly reduces the incidence of venographically detected DVT and PE after arthroplasty.[14,15]

For peer review only

The objective of our study was to compare the 42 day use of dabigatran, nadroparin and rivaroxaban on safety after TKA in a randomised open label pilot study by assessing the risk of major and clinically relevant non-major bleeding using a standardized model of bleeding definitions,[16] in order to obtain insight into the design of a multi-centre study investigating the safety of the new oral anticoagulants for prevention of venous thromboembolism after TKA in the Dutch setting. The rationale of our study was previously published.[17]

For peer review only

Methods

Study design

This study was an open label randomized pilot trial with a three-arm design, conducted at the Martini Hospital in Groningen, The Netherlands. This clinical trial was registered under NCT01431456 at clinicaltrials.gov and opened for enrolment in July 2012. At the start of the study, an independent hospital pharmacist assigned the three treatment groups to a consecutive series of numbers, using a computer-generated randomised list. Before surgery, patients were included and randomly assigned to one of the three treatment groups (dabigatran, rivaroxaban or nadroparin). The allocation concealment was reached by using the sequentially numbered opaque sealed envelopes (SNOSE) method.[18] On enrolment, each patient was assigned to the next consecutive treatment number and the corresponding study medication was dispensed in an open-label fashion.

Ethics statement

This study was approved by the Medical Ethical committee "Regionale Toetsingscommissie Patiëntgebonden Onderzoek" (RTPO) in Leeuwarden, The Netherlands (NL37609.099.12 / RTPO 785).

Patient and public involvement

Patients or the public were not involved in the design, or reporting, or dissemination plans of our research as this study is a pilot study. Patients were involved in the conduct of the trial by sharing experiences during the visits.

Study population

The target population consisted of TKA patients. Patients were recruited from the outpatient orthopaedic surgery clinic at the Martini Hospital in Groningen by the orthopaedic surgeon or trainee. Patients ≥ 18 years and weighing more than 40 kg who were scheduled for primary elective TKA and had provided signed informed consent were eligible for the study. Exclusion criteria included: a known inherited or acquired clinically significant active high risk of bleeding or bleeding disorder; major surgery, trauma, uncontrolled severe arterial hypertension, or

For peer review only

myocardial infarction within the last three months; history of acute intracranial disease or haemorrhagic stroke; gastrointestinal or urogenital bleeding or ulcer disease within the last six months; cirrhotic patients with moderate hepatic impairment (aspartate or alanine aminotransferase levels higher than twice the upper limit of the normal range within the last month); severe renal insufficiency (creatinine clearance < 30 mL/min); other indication for treatment with anticoagulants; active malignant disease; pregnancy or breastfeeding. Patients received standard orthopaedic care and physiotherapy according to the local standardised protocol. During the enrolment of the trial, fast-track knee replacement surgery was implemented.[19,20]

Treatment regimens

Patients were assigned to oral dabigatran etexilate 150 mg or 220 mg (two capsules, 75 mg or 110 mg) once daily, subcutaneous nadroparin 2850 IE = 0.3 ml once daily or 10 mg of oral rivaroxaban once daily. The first dose of dabigatran was one-half of subsequent doses (one capsule, 75 mg or 110 mg). Treatment was continued for a total of 42 days after TKA.

Outcome measures

The primary endpoint of this study was a major bleeding event and or clinically relevant non-major bleeding event within the 42-days treatment period. Any bleeding event that occurred during the 42-days treatment period, perceived by patient, researcher, nurse, orthopaedic surgeon or other health worker were registered and evaluated. A blinded independent expert adjudication committee classified all bleeding events according to international guidelines.[16] A major bleeding was defined as fatal bleeding, clinically overt bleeding associated with a decrease in the haemoglobin level of more than 20 g/l compared with the pre-randomisation level, clinically overt bleeding leading to transfusion of ≥2 units of whole blood or packed cells, critical bleeding (intracerebral, intraocular, intraspinal, pericardial or retroperitoneal), bleeding warranting treatment cessation, bleeding located at the surgical site and leading to re-operation or to any unusual medical intervention or procedure for relief (eg, draining or puncture of a haematoma at the surgical site, transfer to an ICU or emergency room).

For peer review only

Clinically relevant non-major bleeding was defined as spontaneous skin haematoma >25cm^2, wound haematoma >100cm^2, spontaneous nose bleeding or gingival bleeding lasting longer than 5 min, spontaneous rectal bleeding creating more than a spot on toilet paper, macroscopic haematuria either spontaneous or, if associated with an intervention, lasting longer than 24 h, other bleeding events considered clinically relevant by the investigator not qualifying as a major bleeding.

Secondary endpoints of this study were the occurrence of clinical VTE (PE or DVT), compliance, duration of hospital stay, re-hospitalisation, adverse events and Knee injury and Osteoarthritis Outcome Score (KOOS).[21]

The duration of hospital stay and re-hospitalisation were retrieved from electronic medical records.

Compliance was measured by counting the number of doses left over after 42 days of treatment. In our study, patients were deemed compliant if patients didn't miss a single dose.

Adverse events were included in the analysis if the relationship with the study drug was possible, probable or highly probable.

The KOOS (www.koos.nu and supplementary files) is a 42-item self-administered questionnaire that includes five dimensions: pain, disease-related symptoms, activities of daily living function (ADL), sport and recreation function, and knee-related quality of life measured using a Likert scale (0 – 4 scale).[21] The KOOS was administered pre-surgery and six weeks after TKA. A patient with an improvement of ten points or more on a KOOS dimension after TKA was defined as responder.

Statistical analysis

The absolute bleeding risk was estimated as the percentage of patients with a bleed for each treatment group, with the associated 95% confidence intervals. Confidence intervals were derived using the (exact) Clopper-Pearson method. The risk of thromboembolic events was assessed using the same methodology. The analysis was based on the per-protocol population, i.e. all fully compliant patients that were evaluated 42 days after surgery. One-way analysis of variance (ANOVA) was used for analyses of continuous variables between

For peer review only

multiple groups. If normality assumption was violated (Shapiro-Wilk test for normality), the non-parametric Kruskal Wallis test was used. Distribution of nominal variables for different groups was tested for statistical significance using the exact Chi-squared test. All statistical analyses were performed using IBM Statistics SPSS 25.0.0. Due to the explorative nature of this pilot study, all *p*-values will be interpreted as explorative rather than confirmative.[22] The expected sample size for the design of a two-arm clinical trial based on our findings was calculated using the SampleSize4ClinicalTrials package in R (R Studio Version 1.3.959 / R version 4.0.3).

Results

Between July 2012 and November 2013, a total of 198 patients were assessed for eligibility of which 148 patients were randomised to treatment. In total 138 patients were included in the final analysis, see flow diagram (Figure 1). The three treatment groups were well balanced in terms of baseline demographics (Table 1). Of all patients, 63% were woman, with a mean age of 66 years and a mean weight of 89 kg. The mean time from end of surgery to the first study drug intake was 8 hours and 48 minutes. In total, 111 (80%) patients had fast-track TKA. Compared to patients in the other treatment groups, a larger share of patients in the dabigatran group had fast-track TKA. Thirty-three (24%) patients received a patella prosthesis during TKA. Compared to the nadroparin group, a larger share of patients in the rivaroxaban group and smaller share of patients in the dabigatran group received a new patella. Pre-surgery KOOS was well balanced between the three treatment groups.

Primary endpoints

The combined endpoint of major and clinically relevant non-major bleeding was observed in 15 of 45 (33.3%; 95% CI 20.0% – 49.0%) patients who received dabigatran, 11 of 45 (24.4%; 95% CI 12.9% – 39.5%) patients who received nadroparin and 13 of 48 (27.1%; 95% CI 15.3% – 41.8%) patients who received rivaroxaban. There was no significant difference in these bleeding events between dabigatran, rivaroxaban and nadroparin ($p = 0.67$), see Table 2.

For peer review only

Major bleeding events occurred in none of 45 (0%; 95% CI 0% – 7.9%) patients in the dabigatran group, two of 45 (4.4%; 95% CI 0.5% – 15.1%) patients in the nadroparin group and none of 48 (0%; 95% CI 0% – 7.4%) patients in the rivaroxaban group. One of these events was a clinically overt bleeding from the wound leading to transfusion of 2 or more units of whole blood or packed cells and one was adjudicated as a subdural hematoma caused by a fall. There was no difference in major bleeding events between dabigatran, nadroparin and rivaroxaban.

Clinically relevant non-major bleeding events were observed in 15 patients who received dabigatran (33.3%; 95% CI 20.0% – 49.0%), 10 patients who received nadroparine (22.2%; 95% CI 11.2% – 37.1%),) and 13 patients who received rivaroxaban (27.1%; 95% CI 15.3% – 41.8%). Most of these clinically relevant non-major bleeding events were wound hematomas. There was no difference in clinically relevant non-major bleeding events between dabigatran, rivaroxaban and nadroparin. The last major or clinically relevant non-major bleeding event was observed a median seven, seven and six days post-operatively, for dabigatran, nadroparin and rivaroxaban, respectively. In the period between 14 and 42 days after surgery, one major or clinically relevant non-major bleeding event was observed in all treatment groups. The overall risk of major and clinically relevant non-major bleeding was 39 events in a total intention to treat population of 148 randomized patients, i.e. a proportion of 0.26. Using a relative risk difference of 10%, i.e. an absolute difference of 2.6%, we estimated that 4,468 patients per treatment group would be needed in a two-arm non-inferiority design with a statistical power of 80% and a two-sided type I error rate of 2.5%.

For peer review only

Secondary endpoints

The secondary endpoints of this study were: occurrence of clinical VTE, hospital stay, re-admission, adverse events, compliance and KOOS, see Table 2 and 3. Clinical VTE occurred in one of 45 patients receiving dabigatran and in none receiving rivaroxaban or nadroparin. Unfortunately, this patient with VTE developed PE and died. The median duration of hospital stay was three, three and four days for dabigatran, nadroparine and rivaroxaban, respectively. Re-admission within three months post-surgery was not different between treatment groups. The number of patients re-admitted within three months was two (4.4%),

four, (8.9%) and three (6.3%) patients, for dabigatran, nadroparin and rivaroxaban, respectively. Adverse events were reported in nine (20%), two (4.4%) and five (10.4%) patients for dabigatran, nadroparin and rivaroxaban, respectively. Five patients in the dabigatran group (11.1%) experienced gastro-intestinal adverse effects such as abdominal pain, diarrhoea and/or indigestion. In the rivaroxaban group, gastro-intestinal adverse effects were found in two patients (4.4%).

In the dabigatran group, 27 of 44 patients (61.4%) were compliant versus 25 of 44 patients (56.8%) in the nadroparine group and 25 of 48 patients in the rivaroxaban group (52.1%). Compliance was not different between treatment groups. Also, the number of missed days of treatment was not different between treatment groups. In the period between the 42-day follow-up and three months post-surgery, we observed no additional bleeding and VTE events.

The pre- and post-surgery KOOS questionnaires were available for 32 (71%), 35 (77%) and 35 (73%) patients for dabigatran, nadroparin and rivaroxaban, respectively (Table 3). No difference between treatment groups in mean improvement was observed for the KOOS dimensions pain, symptoms, ADL, sport and recreation and quality of life. Also, the proportion of responders, patients with a ten point or more improvement on a KOOS dimension, was not different between dabigatran, nadroparin and rivaroxaban. The standard deviation in KOOS was mostly equal or even higher than the mean.

Discussion

After knee replacement, when bleeding in the replaced joint can have serious consequences, the balance between inducing bleeding and preventing VTE is pivotal in thromboprophylaxis. In this study, we compared bleeding complications of dabigatran, rivaroxaban and nadroparin when used to prevent VTE after TKA surgery. This exploratory study shows that there was no significant difference in major and clinically relevant bleeding between dabigatran, rivaroxaban and nadroparin. The bleeding risk we found is higher than observed in previous knee replacement studies. In such studies a risk of non-major bleeding events of between 1%

For peer review only

and 6.8% was observed.[5,6,23–25] In our study, the risk of bleeding events is higher mainly due to the risk of wound haematomas > 100 cm^2. A possible explanation for the difference in occurrence of clinically relevant non-major bleeding events is our strict adherence to EMA bleeding definitions.[16 24] For instance, in the RECORD 3 and RECORD 4 rivaroxaban trials, surgical site and wound haematomas were not reported.[5,6,8,24] In the EPCAT II trial, extended venous thromboembolism prophylaxis comparing rivaroxaban to aspirin following total hip and knee arthroplasty, only infected wound haematomas were considered clinically relevant.[25] The market authorisation for the prevention of venous thromboembolism after total knee arthroplasty for dabigatran and rivaroxaban is 10-14 days. In our study, patients received thromboprophylaxis for 42 days. Most bleeding events occurred during the first 14 days post-surgery. Notably, bleeding risks after the first 14 days use of dabigatran, nadroparin and rivaroxaban were low and not different between treatment groups. Based on our pilot-study, a meta-analysis of dabigatran and rivaroxaban in TKA and the EPCAT II trial, it is expected that large numbers of patients have to be included in a clinical trial to detect a clinically relevant difference in major and clinically relevant non-major bleeding between NOACs and LMWHs when used for 42 days.[8,25] Therefore, costs of conducting a multicentre clinical trial based on the evaluated protocol and a formal power calculation are expected to be very high.

As expected, the number of symptomatic VTE cases was very low. Unfortunately, one case of PE occurred in the dabigatran group, leading to the death of the patient.

It is generally assumed that patients would prefer the oral route to subcutaneous injections. However, it is unknown if the route of administration has an impact on patient compliance. In our pilot-study, we found no clear indication for a difference in compliance between rivaroxaban, nadroparin and dabigatran.

Although we found no differences in the number of patients with adverse reactions, the number of patients with gastro-intestinal adverse reaction in the dabigatran group appeared to be higher than in the other treatment groups. Dabigatran is also in other clinical trials associated with gastro-intestinal adverse effects.[26] Between dabigatran, nadroparin and

only

rivaroxaban no difference in patient reported outcomes as assessed with the KOOS was found. The hypothesis was that joint haemorrhages could impair subjective improvement after TKA as assessed by KOOS. Our pilot study shows that it is feasible to assess KOOS in a study evaluating bleeding complications of thromboprophylaxis. However, variability in KOOS is probably too high to detect differences in KOOS in a future multicentre clinical trial.

The results of this pilot study should be interpreted with caution due to some limitations. Firstly, as a pilot-study, the size of the study population is limited. Although our aim was to conduct a pilot study to design a multicentre study with an improved external validity compared to available randomised clinical trials, we had to exclude a large number of patients some of who will receive thromboprophylaxis with NOACs in regular clinical practice. Additionally, we had to exclude patients with a high risk of bleeding who may receive thromboprophylaxis with NOACs in regular clinical practice.

For peer review only

Conclusion

A multicentre clinical trial may be feasible based on the recruitment speed and satisfaction with the trial protocol. No differences in major and non-major clinically relevant bleeding events were found in a pilot-study of the head-to-head comparison of dabigatran, nadroparin and rivaroxaban as 42-day thromboprophylaxis in TKA. After the first 14 days use of dabigatran, nadroparin or rivaroxaban, bleeding risks were low and not different. This finding implies that conducting a multicentre trial will require substantial investment to achieve an adequate number of evaluated patients. Furthermore, due to the high variability in the KOOS, this outcome score may not be suitable to detect functional loss due to bleeding in a future clinical trial.

Acknowledgements

The adjudication committee (mrs A. van der Aart, hospital pharmacist; mrs A. van der Velden, internist-haematologist / oncologist and dr. R.W. Brouwer, orthopaedic surgeon) is thanked for the careful assessment of bleeding events. Dr Brouwer joint the writing committee after adjudication of bleeding events. Mrs Roya Sorghabi is thanked for clinical trial management

and data acquisition. Nurses and doctors of the orthopaedic surgery department are thanked for screening patients for bleeding events.

Author Contributions

LV, MH, CG and JR conceived the idea of the study and were responsible for the design of the study. NV, LV and MH performed the statistical analysis for the study. The initial draft of the manuscript was prepared by LV, MS, MH, JR, RB and NV and then circulated repeatedly among all authors for critical revision. LV and MS were responsible for the acquisition of the data and MH and NV contributed to the interpretation of the results. LV was the coordinator of the study and lead writer. All authors helped plan the study, evolve analysis plans and critically revise successive drafts of the manuscript.

Funding

This research received no specific grant from any funding agency in the public, commercial or not-for-profit sectors.

Competing interests

MH reports grants from Bayer and personal fees from Boehringer Ingelheim during the conduct of the study. Other authors have nothing to report.

Data sharing

Data can made be available upon request to the corresponding author.

For peer review only

Table 1. Patient characteristics

	Dabigatran n = 45	Nadroparine n = 45	Rivaroxaban n = 48	Total n = 138
Female n (%)	26 (58%)	31 (69%)	30 (63%)	87 (63%)
Age (year) [±SD]	66±7.9	67±10	65±7.5	66±8.7
Weight (kg) [±SD]	88±12	89±18	90±21	89±17
Fast-track surgery (%)	41 (91%)	35 (78%)	35 (73%)	111 (80%)
Received new patella (%)	7 (16%)	10 (22%)	16 (33%)	33 (24%)
History of VTE (%)	1 (2.2%)	1 (2.2%)	2 (4.2%)	4 (2.9%)
Time to first dose (h) [±SD]	8.6±2.3	9.0±2.2	8.8±2.1	8.8±2.2
Pre-surgery KOOS [±SD]				
- pain {n}	47±15 {32}	48±20 {35}	51±21 {35}	49±19 {102}
- symptoms {n}	55±16 {32}	49±18 {35}	50±18 {35}	51±17 {102}
- ADL {n}	52±15 {32}	48±19 {35}	56±19 {35}	52±18 {102}
- sport and recreation {n}	23±25 {29}	24±28 {31}	22±22 {29}	23±25 {89}
- quality of life {n}	30±12 {31}	30±16 {35}	30±15 {35}	30±14 {101}

ADL: activities of daily living function; KOOS: Knee injury and Osteoarthritis Outcome Score; SD: standard deviation; VTE: venous thromboembolism.

For peer review only

Table 2. Primary and secondary outcomes of thromboprophylaxis with dabigatran, nadroparin or rivaroxaban after total knee arthroplasty

	Dabigatran n = 45		Nadroparin n = 45		Rivaroxaban n = 48		*p*-value
	n	%	*n*	%	*n*	%	
Primary outcome							
Major and clinically relevant non-major bleeding	15	33.3	11	24.4	13	27.1	$p = 0.67$
Major bleedings	0	0	2	4.4	0	0	$p = 0.21$
Fatal bleeding	0	0	0	0	0	0	
Clinically overt bleeding, Hb decrease>20 g/l	0	0	0	0	0	0	
Clinically overt bleeding, transfusion	0	0	1	2.2	0	0	$p = 0.65$
Critical bleeding	0	0	1*	2.2	0	0	$p = 0.65$
Bleeding warranting treatment cessation	0	0	1*	2.2	0	0	$p = 0.65$
Bleeding located at surgical site	0	0	0	0	0	0	
Clinically relevant non-major bleeding	15	33.3	10	22.2	13	27.1	$p = 0.51$
Spontaneous skin haematoma >25 cm2	1	2.2	0	0	0	0	$p = 0.65$
Wound haematoma >100 cm2	14	31.1	9	20.0	12	25.0	$p = 0.49$
Spontaneous nose bleeding > 5 min	0	0	1	2.2	0	0	$p = 0.65$
Spontaneous rectal bleeding > 1 spot	0	0	0	0	0	0	
Macroscopic haematuria > 24 h	0	0	0	0	1	2.1	$p = 1.0$
Other bleeding event (not major)	0	0	0	0	0	0	
Last primary outcome (median days after surgery) [range]	7 [1 – 21]		7 [2 – 26]		6 [2 – 16]		$p = 0.32$
Patients with primary outcome more than 14 days after surgery (%)	1	2.2	1	2.2%	1	2.1	$p = 1.0$
Secondary outcome							
VTE	1*	2.2	0	0	0	0	$p = 0.65$
Death due to VTE	1*		0		0		
DVT	0		0		0		
PE	1*		0		0		
Hospital stay (median, days) [range]	3 [2 – 9]		3 [2 – 10]		4 [2 – 28]		$p = 0.098$
Re-admission within three months (%)	2 (4.4%)		4 (8.9%)		3 (6.3%)		$p = 0.77$
Patients with adverse reactions (%)	9 (20.0%)		2 (4.4%)		5 (10.4%)		$p = 0.072$
Number of patient compliant (%)	27 of 44 (61.4%)†		25 of 44 (56.8%)†		25 (52.1%)		$p = 0.66$
Days of missed dosages (median, days) [range]	0 [0 – 7]; n=42		0 [0 – 23]; n=40		0 [0 – 4]; n=40		$p = 0.73$

VTE: venous thromboembolism; PE: pulmonary embolism; DVT: deep venous thrombosis; SD: standard deviation;
*identical patient; † one missing value. Due to the explorative nature of this pilot study, all p-values are explorative rather than confirmative and are not corrected for multiple testing.

For peer review only

Table 3. Knee injury and Osteoarthritis Outcome Score (KOOS) for patients with dabigatran, nadroparin or rivaroxaban thromboprophylaxis after total knee arthroplasty. Difference between six weeks post-surgery and pre-surgery KOOS and proportion of patients with 10 points or more improvement in KOOS between six weeks post-surgery and pre-surgery.

	Dabigatran	Nadroparin	Rivaroxaban	*p*-value
Pain	n = 32	n = 35	n = 35	
Difference six weeks post-surgery and pre-surgery (mean±SD)	25±26	19±22	22±23	p=0.51
Patients with ≥10 points improvement post- vs pre-surgery (n; %)	22; 69%	26; 74%	27; 77%	p=0.78
Symptoms	n = 32	n = 35	n = 35	
Difference six weeks post-surgery and pre-surgery (mean±SD)	12±20	14±20	16±19	p=0.63
Patients with ≥10 points improvement post- vs pre-surgery (n, %)	17; 53%	20; 57%	22; 63%	p=0.72
ADL	n = 32	n = 35	n = 35	
Difference six weeks post-surgery and pre-surgery (mean±SD)	25±20	23±21	21±17	p=0.75
Patients with ≥10 points improvement post- vs pre-surgery (n, %)	23; 72%	25; 71%	27; 77%	p=0.88
Sport and recreation	n = 29	n = 31	n = 29	
Difference six weeks post-surgery and pre-surgery (mean±SD)	24±38	25±41	28±33	p=0.89
Patients with ≥10 points improvement post- vs pre-surgery (n, %)	17; 59%	21; 68%	23;79%	p=0.24
Quality of Life	n = 31	n = 35	n = 35	
Difference six weeks post-surgery and pre-surgery (mean±SD)	26±27	18±20	22±21	p=0.39
Patients with ≥10 points improvement post- vs pre-surgery (n, %)	24;77%	23;66%	23; 66%	p=0.55

ADL: activities of daily living function; SD: standard deviation. Due to the explorative nature of this pilot study, all p-values are explorative rather than confirmative and are not corrected for multiple testing.

For peer review only - http://bmjopen.bmj.com/site/about/guidelines.xhtml

Figure 1. Flow diagram. AF: atrial fibrillation; ALT: alanine aminotransferase; AST: aspartate aminotransferase.

For peer review only

Reference list

1. Warwick D, Friedman RJ, Agnelli G, et al. Insufficient duration of venous thromboembolism prophylaxis after total hip or knee replacement when compared with the time course of thromboembolic events: findings from the Global Orthopaedic Registry. *The Journal of bone and joint surgery British volume* 2007; **89**(6): 799-807.
2. White RH. The epidemiology of venous thromboembolism. *Circulation* 2003; **107**(23 Suppl 1): I4-8.
3. Falck-Ytter Y, Francis CW, Johanson NA, et al. Prevention of VTE in orthopedic surgery patients: Antithrombotic Therapy and Prevention of Thrombosis, 9th ed: American College of Chest Physicians Evidence-Based Clinical Practice Guidelines. *Chest* 2012; **141**(2 Suppl): e278S-325S.
4. Rachidi S, Aldin ES, Greenberg C, Sachs B, Streiff M, Zeidan AM. The use of novel oral anticoagulants for thromboprophylaxis after elective major orthopedic surgery. *Expert review of hematology* 2013; **6**(6): 677-95.
5. Lassen MR, Ageno W, Borris LC, et al. Rivaroxaban versus enoxaparin for thromboprophylaxis after total knee arthroplasty. *The New England journal of medicine* 2008; **358**(26): 2776-86.
6. Turpie AG, Lassen MR, Davidson BL, et al. Rivaroxaban versus enoxaparin for thromboprophylaxis after total knee arthroplasty (RECORD4): a randomised trial. *Lancet* 2009; **373**(9676): 1673-80.
7. Turpie AG, Haas S, Kreutz R, et al. A non-interventional comparison of rivaroxaban with standard of care for thromboprophylaxis after major orthopaedic surgery in 17,701 patients with propensity score adjustment. *Thrombosis and haemostasis* 2014; **111**(1): 94-102.
8. Gomez-Outes A, Terleira-Fernandez AI, Suarez-Gea ML, Vargas-Castrillon E. Dabigatran, rivaroxaban, or apixaban versus enoxaparin for thromboprophylaxis after total hip or knee replacement: systematic review, meta-analysis, and indirect treatment comparisons. *BMJ* 2012; **344**: e3675.
9. Huisman MV, Quinlan DJ, Dahl OE, Schulman S. Enoxaparin versus dabigatran or rivaroxaban for thromboprophylaxis after hip or knee arthroplasty: Results of separate pooled analyses of phase III multicenter randomized trials. *Circulation Cardiovascular quality and outcomes* 2010; **3**(6): 652-60.
10. Friedman RJ, Dahl OE, Rosencher N, et al. Dabigatran versus enoxaparin for prevention of venous thromboembolism after hip or knee arthroplasty: a pooled analysis of three trials. *Thrombosis research* 2010; **126**(3): 175-82.
11. Venker BT, Ganti BR, Lin H, Lee ED, Nunley RM, Gage BF. Safety and Efficacy of New Anticoagulants for the Prevention of Venous Thromboembolism After Hip and Knee Arthroplasty: A Meta-Analysis. *The Journal of arthroplasty* 2017; **32**(2): 645-52.
12. Vereniging NI. Keuze en duur van tromboseprofylaxe bij grote orthopedische en traumatologische ingrepen. https://richtlijnendatabase.nl/richtlijn/antitrombotisch_beleid/preventie_vte/keuze_en_duur_profylaxe_grote_ingrepen.html#verantwoording (accessed 13-02-2018).
13. Ettema HB, Mulder MC, Nurmohamed MT, Buller HR, Verheyen CC. Dutch orthopedic thromboprophylaxis: a 5-year follow-up survey. *Acta orthopaedica* 2009; **80**(1): 109-12.
14. White RH, Romano PS, Zhou H, Rodrigo J, Bargar W. Incidence and time course of thromboembolic outcomes following total hip or knee arthroplasty. *Archives of internal medicine* 1998; **158**(14): 1525-31.

For peer review only

15. Bergqvist D, Benoni G, Bjorgell O, et al. Low-molecular-weight heparin (enoxaparin) as prophylaxis against venous thromboembolism after total hip replacement. *The New England journal of medicine* 1996; **335**(10): 696-700.
16. (EMA) EMA. Guideline on clinical investigation of medicinal products for the prophylaxis of high intra- and postoperative venous thromboembolic risk. *CPMP/EWP/707/98* 2007; **15-11-2007**.
17. Veen L, van Raay JJ, Gerritsma-Bleeker CL, Veeger NJ, Hulst M. Direct treatment comparison of DAbigatran and RIvaroxaban versus NAdroparin in the prevention of venous thromboembolism after total knee arthroplasty surgery: design of a randomised pilot study (DARINA). *BMJ open* 2013; **3**(1).
18. Doig GS, Simpson F. Randomization and allocation concealment: a practical guide for researchers. *Journal of critical care* 2005; **20**(2): 187-91; discussion 91-3.
19. Husted H, Jensen CM, Solgaard S, Kehlet H. Reduced length of stay following hip and knee arthroplasty in Denmark 2000-2009: from research to implementation. *Archives of orthopaedic and trauma surgery* 2012; **132**(1): 101-4.
20. Van Egmond JC, Verburg H, Mathijssen NM. The first 6 weeks of recovery after total knee arthroplasty with fast track. *Acta orthopaedica* 2015; **86**(6): 708-13.
21. de Groot IB, Favejee MM, Reijman M, Verhaar JA, Terwee CB. The Dutch version of the Knee Injury and Osteoarthritis Outcome Score: a validation study. *Health and quality of life outcomes* 2008; **6**: 16.
22. Julious SA. Sample size of 12 per group rule of thumb for a pilot study. *Pharmaceutical Statistics* 2005; **4**(4): 5.
23. Eriksson BI, Dahl OE, Rosencher N, et al. Oral dabigatran etexilate vs. subcutaneous enoxaparin for the prevention of venous thromboembolism after total knee replacement: the RE-MODEL randomized trial. *Journal of thrombosis and haemostasis : JTH* 2007; **5**(11): 2178-85.
24. Dahl OE, Quinlan DJ, Bergqvist D, Eikelboom JW. A critical appraisal of bleeding events reported in venous thromboembolism prevention trials of patients undergoing hip and knee arthroplasty. *Journal of thrombosis and haemostasis : JTH* 2010; **8**(9): 1966-75.
25. Anderson DR, Dunbar M, Murnaghan J, et al. Aspirin or Rivaroxaban for VTE Prophylaxis after Hip or Knee Arthroplasty. *The New England journal of medicine* 2018; **378**(8): 699-707.
26. Connolly SJ, Ezekowitz MD, Yusuf S, et al. Dabigatran versus warfarin in patients with atrial fibrillation. *The New England journal of medicine* 2009; **361**(12): 1139-51.

For peer review only

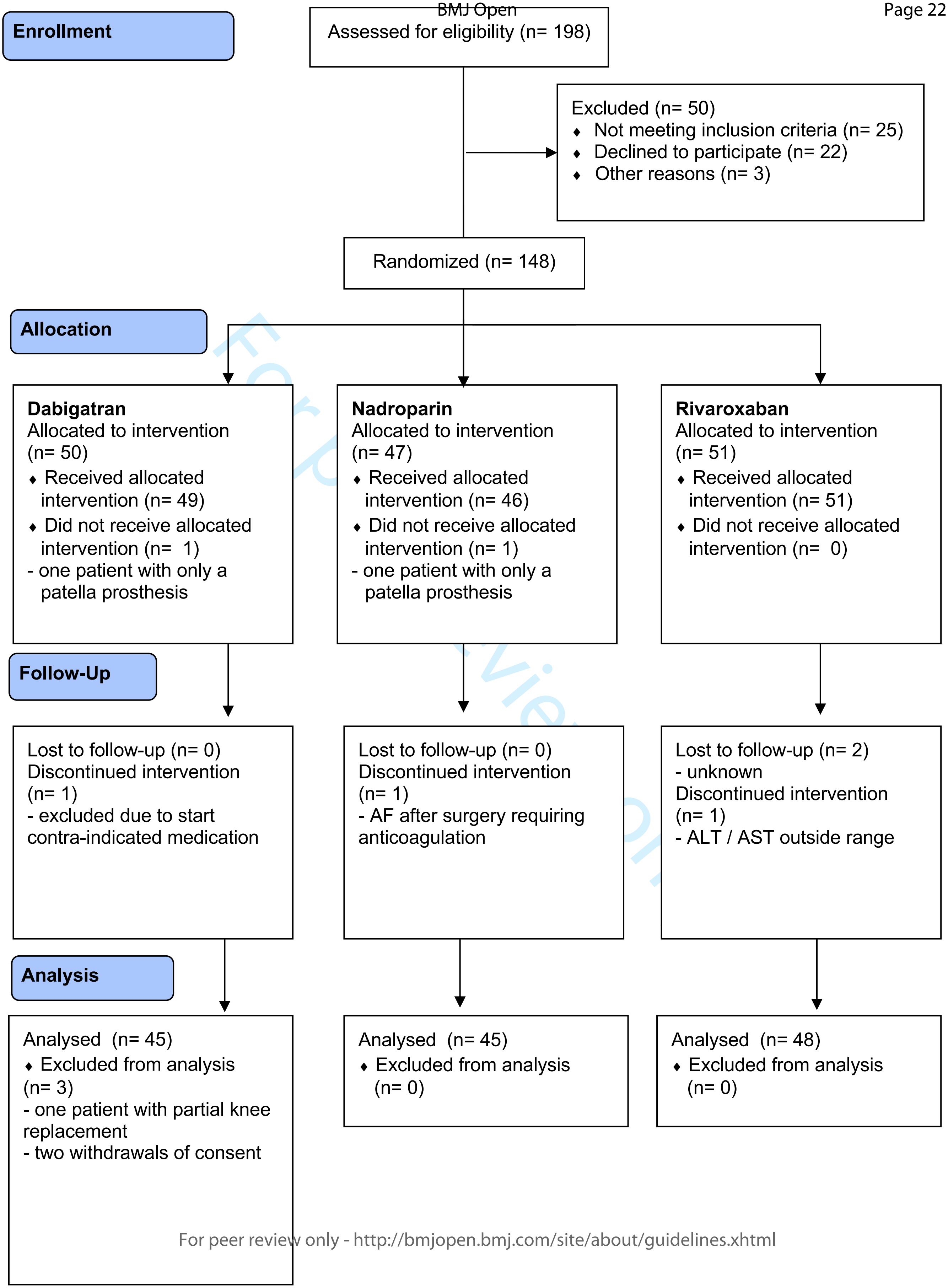

For peer review only - http://bmjopen.bmj.com/site/about/guidelines.xhtml

Naam: Geb.datum: Datum:

Knee injury and Osteoarthritis Outcome Score (KOOS)

Groot IB de, Favejee M, Reijman M, Verhaar JAN, Terwee CB.

Instructies: Deze enquête stelt vragen in verband met uw visie betreffende uw knie. Deze informatie helpt ons te achterhalen hoe u zich voelt en in hoeverre het mogelijk is voor u om uw dagelijkse activiteiten uit te voeren. Beantwoord de onderstaande vragen door **één** antwoord aan te vinken dat voor u van toepassing is. Als u niet geheel zeker bent van uw antwoord, graag toch het best mogelijke antwoord geven.

Belangrijk: Probeer uitsluitend antwoord met betrekking tot het kniegewricht waarvoor u in deze studie behandeld wordt.

For peer review only

Symptomen
Deze vragen dienen te worden beantwoord met betrekking tot de knie symptomen gedurende de afgelopen week.

	Nooit	Bijna nooit	Soms	Vaak	Altijd
**S1. Is uw knie gezwollen?**	O	O	O	O	O
**S2. Voelt u knarsen in uw knie of hoort u uw knie klikken of een ander geluid als u uw knie beweegt?**	O	O	O	O	O
**S3. Blijft uw knie steken of schiet uw knie op slot bij bewegen?**	O	O	O	O	O

	Altijd	Vaak	Soms	Bijna nooit	Nooit
**S4. Kan u uw knie volledig strekken?**	O	O	O	O	O
**S5. Kan u uw knie volledig buigen?**	O	O	O	O	O

Stijfheid
De volgende vragen betreffen de hoeveelheid gewrichtstijfheid die u ervaren heeft in uw knie gedurende de **afgelopen week.** Stijfheid is een gevoel van restrictie of traagheid in de gemakkelijkheid waarmee u uw kniegewricht kan bewegen.

	Niet	Mild	Gemiddeld	Ernstig	Extreem
**S6. Hoe ernstig is de stijfheid van uw kniegewricht als u wakker wordt 's morgens?**	O	O	O	O	O
**S7. Hoe ernstig is de stijfheid van uw kniegewricht nadat u gezeten, gelegen of gerust heeft later op de dag?**	O	O	O	O	O

**Pijn**	Nooit	Maandelijks	Wekelijks	Dagelijks	Altijd
**P1. Hoe vaak ervaart u kniepijn?**	O	O	O	O	O

Hoeveel kniepijn heeft u ervaren de **afgelopen week** tijdens de volgende activiteiten?

	Niet	Mild	Gemiddeld	Ernstig	Extreem

Naam: Geb.datum: Datum:

**P2. Draaien / roteren van uw knie**	O	O	O	O	O
**P3. Volledig strekken van de knie**	O	O	O	O	O
**P4. Volledig buigen van de knie**	O	O	O	O	O
**P5. Lopen op een vlak oppervlak**	O	O	O	O	O
**P6. Op- en neer lopen van een trap**	O	O	O	O	O
**P7. ‘s Nachts in bed**	O	O	O	O	O
**P8. Zitten of liggen**	O	O	O	O	O
**P9. Rechtop staan**	O	O	O	O	O

Functioneren, dagelijkse bezigheden

De volgende vragen betreffen uw fysieke functioneren. Hierbij wordt bedoeld de mogelijkheid om u te bewegen en voor uzelf te zorgen. Voor de volgende activiteiten graag aangeven wat de moeilijkheidsgraad is die u ervaren heeft gedurende de afgelopen week als gevolg van uw knie.

	**Niet**	**Mild**	**Gemiddeld**	**Ernstig**	**Extreem**
**A1. Aflopen van een trap**	O	O	O	O	O
**A2. Oplopen van een trap**	O	O	O	O	O
**A3. Opstaan nadat u gezeten heeft**	O	O	O	O	O
**A4. Staan**	O	O	O	O	O
**A5. Buigen om iets op te pakken**	O	O	O	O	O
**A6. Lopen op een vlak oppervlak**	O	O	O	O	O
**A7. In- en uit een auto stappen**	O	O	O	O	O
**A8. Boodschappen doen / winkelen**	O	O	O	O	O
**A9. Sokken / panty aandoen**	O	O	O	O	O
**A10. Opstaan uit bed**	O	O	O	O	O
**A11. Sokken / panty uitdoen**	O	O	O	O	O
**A12. In bed liggen (draaien, behouden van de knie positie)**	O	O	O	O	O
**A13. In- en uit bad stappen**	O	O	O	O	O
**A14. Zitten**	O	O	O	O	O
**A15. Naar het toilet gaan**	O	O	O	O	O

For peer review only

For peer review only - http://bmjopen.bmj.com/site/about/guidelines.xhtml

Naam: Geb.datum: Datum:

**A16. Zware huishoudelijke taken (verplaatsen van dozen, boenen van vloer, etc.)**	O	O	O	O	O
**A17. Lichte huishoudelijke taken (koken, stoffen, etc.)**	O	O	O	O	O

Functioneren, sporten, en ontspanningsactiviteiten

De volgende vragen betreffen uw fysieke functies wanneer u actief bent op een hoger niveau. De vragen dienen te worden beantwoord naar aanleiding van de moeilijkheidsgraad die u ervaart door uw knie gedurende de **afgelopen week**.

	**Niet**	**Mild**	**Gemiddeld**	**Ernstig**	**Extreem**
**SP1. Hurken**	O	O	O	O	O
**SP2. Rennen**	O	O	O	O	O
**SP3. Springen**	O	O	O	O	O
**SP4. Draaien / roteren van uw geblesseerde knie**	O	O	O	O	O
**SP5. Knielen / bukken**	O	O	O	O	O

For peer review only

Kwaliteit van leven

	**Nooit**	**Maandelijks**	**Wekelijks**	**Dagelijks**	**Altijd**
**Q1. Hoe vaak bent u zich bewust van uw knie probleem?**	O	O	O	O	O

	**Niet**	**Nauwelijks**	**Gemiddeld**	**Ernstig**	**Extreem**
**Q2. Heeft u uw levenswijze aangepast om potentiële schadelijke activiteiten aan u knie te vermijden?**	O	O	O	O	O
**Q3. In welke mate bent u gehinderd door het gemis van vertrouwen in uw knie?**	O	O	O	O	O

	**Niet**	**Mild**	**Gemiddeld**	**Ernstig**	**Extreem**
**Q4. In het algemeen, hoeveel hinder ervaart u door uw knie?**	O	O	O	O	O

Scoring: elk item wordt gescoord tussen 0 en 4 en de ruwe score van elke sectie is de som of de item scores. De score wordt daarna omgezet in een 0 -100 schaal. Een hogere score indiceert minder problemen

**Schaal**	**Ruwe score**	**Herberekende score**	**MDC90**
Pijn	/36	100 - Actueel ruwe score x 100 : Mogelijke ruwe score range	12 punten
Symptomen	/28		8 punten
ADL	/68		10 punten
Sport/Rec	/20		19 punten
QvL	/16		13 punten

Reporting checklist for randomised trial.

Based on the CONSORT guidelines.

Instructions to authors

Complete this checklist by entering the page numbers from your manuscript where readers will find each of the items listed below.

Your article may not currently address all the items on the checklist. Please modify your text to include the missing information. If you are certain that an item does not apply, please write "n/a" and provide a short explanation.

Upload your completed checklist as an extra file when you submit to a journal.

In your methods section, say that you used the CONSORTreporting guidelines, and cite them as:

Schulz KF, Altman DG, Moher D, for the CONSORT Group. CONSORT 2010 Statement: updated guidelines for reporting parallel group randomised trials

		Reporting Item	Page Number
**Title and Abstract**			
Title	#1a	Identification as a randomized trial in the title.	Page 1
Abstract	#1b	Structured summary of trial design, methods, results, and conclusions	Page 2
**Introduction**			
Background and objectives	#2a	Scientific background and explanation of rationale	Page 4
Background and objectives	#2b	Specific objectives or hypothesis	Page 4
**Methods**			
Trial design	#3a	Description of trial design (such as parallel, factorial) including allocation ratio.	Page 6
Trial design	#3b	Important changes to methods after trial commencement (such as eligibility criteria), with reasons	Page 6

For peer review only

Participants	#4a	Eligibility criteria for participants	Page 6
Participants	#4b	Settings and locations where the data were collected	Page 6
Interventions	#5	The experimental and control interventions for each group with sufficient details to allow replication, including how and when they were actually administered	Page 6 – 7
Outcomes	#6a	Completely defined prespecified primary and secondary outcome measures, including how and when they were assessed	Page 7
Sample size	#7a	How sample size was determined.	Page 8
Sample size	#7b	When applicable, explanation of any interim analyses and stopping guidelines	Page 8
Randomization - Sequence generation	#8a	Method used to generate the random allocation sequence.	
Page 6			
Randomization - Sequence generation	#8b	Type of randomization; details of any restriction (such as blocking and block size)	
Page 6			
Randomization - Allocation concealment mechanism	#9	Mechanism used to implement the random allocation sequence (such as sequentially numbered containers), describing any steps taken to conceal the sequence until interventions were assigned	Page 6
Randomization - Implementation	#10	Who generated the allocation sequence, who enrolled participants, and who assigned participants to interventions	Page 6
Blinding	#11a	If done, who was blinded after assignment to interventions (for example, participants, care providers, those assessing outcomes) and how.	Page 6
Blinding	#11b	If relevant, description of the similarity of interventions	Page 6
Statistical methods	#12a	Statistical methods used to compare groups for primary and secondary outcomes	Page 8
Statistical methods	#12b	Methods for additional analyses, such as subgroup analyses and adjusted analyses	Page 8

For peer review only

Outcomes	#6b	Any changes to trial outcomes after the trial commenced, with reasons	Page 7
**Results**			
Participant flow diagram (strongly recommended)	#13a	For each group, the numbers of participants who were randomly assigned, received intended treatment, and were analysed for the primary outcome	Figure 1
Participant flow	#13b	For each group, losses and exclusions after randomization, together with reason	Figure 1
Recruitment	#14a	Dates defining the periods of recruitment and follow-up	Page 8
Recruitment	#14b	Why the trial ended or was stopped	n/a
Baseline data	#15	A table showing baseline demographic and clinical characteristics for each group	Page 15, table 1.
Numbers analysed	#16	For each group, number of participants (denominator) included in each analysis and whether the analysis was by original assigned groups	Figure 1
Outcomes and estimation	#17a	For each primary and secondary outcome, results for each group, and the estimated effect size and its precision (such as 95% confidence interval)	Page 9
Outcomes and estimation	#17b	For binary outcomes, presentation of both absolute and relative effect sizes is recommended	Page 9
Ancillary analyses	#18	Results of any other analyses performed, including subgroup analyses and adjusted analyses, distinguishing pre-specified from exploratory	Page 9 – 11
Harms	#19	All important harms or unintended effects in each group (For specific guidance see CONSORT for harms)	Page 10
**Discussion**			
Limitations	#20	Trial limitations, addressing sources of potential bias, imprecision, and, if relevant, multiplicity of analyses	Page 11
Interpretation	#22	Interpretation consistent with results, balancing benefits and harms, and considering other relevant evidence	Page 11
Registration	#23	Registration number and name of trial registry	Page 1

For peer review only

Other Information

Protocol	#24	Where the full trial protocol can be accessed, if available	Page 5 (ref 17) and Appendix
Funding	#25	Sources of funding and other support (such as supply of drugs), role of funders	Page 13

Notes:

- 15: Page 15, table 1.
- 18: Page 9 – 11
- 24: Page 5 (ref 17) and Appendix The CONSORT checklist is distributed under the terms of the Creative Commons Attribution License CC-BY. This checklist was completed on 24. April 2020 using https://www.goodreports.org/, a tool made by the EQUATOR Network in collaboration with Penelope.ai

For peer review only
